# Mechanistic insights from metagenomics into the early-stage quality improvement of licorice under partial replacement of chemical by organic fertilizers

**DOI:** 10.3389/fpls.2025.1613771

**Published:** 2025-06-25

**Authors:** Jiancai Xiao, Pengchao Gao, Kunyang Lai, Yan Binbin, Sheng Wang, Yuping Zhao, Xiufu Wan, Chaogeng Lyu, Chuanzhi Kang, Lanping Guo, Yan Zhang

**Affiliations:** ^1^ National Key Laboratory for Quality Ensurance and Sustainable Use of Dao-di Herbs, National Resource Center for Chinese Materia Medica, China Academy of Chinese Medical Sciences, Beijing, China; ^2^ School of Traditional Chinese Medicine, Guangdong Pharmaceutical University, Guangzhou, China

**Keywords:** licorice, metagenomics, rational fertilization, microbiology, quality, soil

## Abstract

Substituting chemical fertilizers with organic alternatives has gained increasing attention for its potential to enhance crop yield, quality, and soil health. In the early stages of licorice cultivation, the short-term effects of organic fertilization on microbial community dynamics, soil properties, and bioactive compound accumulation remain insufficiently understood. A metagenomic approach was applied to analyze microbial compositions in both bulk and rhizosphere soils under different fertilization treatments. Results showed that organic fertilization significantly increased licorice yield and the accumulation of bioactive compounds compared to chemical fertilization. Full organic substitution improved soil nutrient content, organic matter, and carbon levels while reducing enzyme activities such as urease and protease. Additionally, organic fertilization promoted bacterial diversity, with notable increases in Actinobacteria and fungal taxa such as Ascomycota and Basidiomycota. Correlations between soil microbial communities, enzyme activity, and secondary metabolite synthesis—particularly glycyrrhizic acid—were observed. These findings suggest that organic fertilization fosters microbial diversity and soil health, ultimately benefiting licorice production and quality in the early stages of cultivation while contributing to sustainable agricultural practices.

## 1 Introduction

Over the past two decades, the intensification of agricultural systems in China has not proportionally increased plant productivity despite the significant rise in fertilizer application (Jiao et al., 2016; Wang et al., 2020a). Instead, this has led to low nutrient utilization efficiency, reduced crop quality, and serious environmental pollution, presenting a series of barriers to sustainable development (Maltas et al., 2018), particularly evident in the cultivation and efficacy of medicinal herbs (Deng et al., 2024). Traditionally, we attributed this trend to inadequate management of biological processes occurring in the rhizosphere, which determines crop nutrient utilization and allows for early effects of fertilizers (Zhang et al., 2010). Controlling rhizosphere processes was considered an effective strategy to enhance nutrient use efficiency and crop productivity, garnering widespread attention (Zhang et al., 2018). However, the primary source of rhizosphere microbiota is adjacent soil, and changes in microbial communities in the bulk soil directly impact the rhizosphere community, despite lower nutrient conditions, nutrient transformation rates, and microbial activity in the bulk soil (Hartmann et al., 2008; Ai et al., 2012; Han et al., 2023). A comprehensive understanding of fertilizer strategies’ impact on soil hotspots and highly active microenvironments, such as the rhizosphere microenvironment providing essential services for human well-being, along with the involved mechanisms, is crucial for adopting optimal conservation strategies.

Soil microorganisms perform various ecological functions (Mendes et al., 2014), including nutrient cycling through crop residue decomposition (Yu et al., 2024), promoting crop growth (Pii et al., 2015), enhancing medicinal herb quality (Wang et al., 2020b), and resisting pathogens (Strickland and Rousk, 2010; Maier et al., 2018). However, the soil microbial community is highly regulated by changes in soil physicochemical properties such as pH (Zhalnina et al., 2015), electrical conductivity, organic matter, nitrogen content, etc (Ren et al., 2021). Additionally, the functional capacity of soil microbial communities is reflected in the activity of enzymes involved in nutrient mineralization processes (Miao et al., 2019). For instance, soil microorganisms produce proteases, which are hydrolytic enzymes that break down proteins into amino acids, releasing nitrogen elements (Liu et al., 2017). Moreover, they also produce enzymes related to microbial phosphorus acquisition, including alkaline phosphatase and acid phosphatase, as well as enzymes related to carbon acquisition, such as cellulase and glucose dehydrogenase (Tao et al., 2015). Simultaneously, soil microorganisms act as the plant’s second genome, closely related to the quality of medicinal plants (Liu et al., 2024). They primarily promote plant growth by providing nutrients, enhancing nutrient bioavailability, and increasing plant nutrient acquisition capacity (N, P, K, etc.) (Zhang et al., 2021; Huang et al., 2020; Luo et al., 2018a; Qaswar et al., 2020). For example, nitrogen-fixing bacteria like Rhizobium and mycorrhizal fungi contribute to nitrogen fixation (Xu and Wang, 2023), while *Bacillus* spp. contribute to phosphorus solubilization (Lu et al., 2023). Reported instances include soil microorganisms regulating gene expression related to secondary metabolite synthesis in medicinal plants, and synthesizing key enzymes for precursor synthesis of active plant components, such as *Bacillus pumilus* enhancing Glycyrrhizic acid synthesis pathways in licorice (Xie et al., 2019). In summary, understanding the interplay between microbial-mediated medicinal plants, soil physicochemical properties, microbial communities, and their relationship with enzyme activity may help identify key drivers of soil nutrient effectiveness (Chen et al., 2020). This understanding could explain the intrinsic environmental drivers of medicinal plant quality formation and growth development and enable targeted control measures.

A substantial body of experiments has confirmed that microbial diversity, enzyme activity, soil properties, and plant growth processes, including secondary metabolisms, are significantly influenced by fertilizer management (Wu et al., 2017). Substituting chemical fertilizers with organic fertilizers has been demonstrated as a promising strategy to improve soil fertility and medicinal plant productivity. For instance, researchers demonstrated that organic fertilizers replacing chemical fertilizers enhanced soil organic carbon and the effectiveness of quick nutrients, significantly increasing the yield of *Centella asiatica* while notably boosting asiaticoside content (Siddiqui et al., 2011). Similarly, Xiang et al. (2020) put forward their perspective, observing increased soil fertility, fungal abundance, diversity, and dominant microbial communities when combining nitrogen-phosphorus-potassium compound fertilizers with organic fertilizers. Additionally, although previous studies mainly focused on long-term field experiments, some research has confirmed that organic fertilizers, even in a single growth process, can have beneficial effects on crop productivity and secondary metabolism (Lazcano et al., 2013), indicating short-term mechanisms of organic fertilizer action that may contribute to plant growth promotion. Moreover, comprehensive information is lacking regarding the overall impact of organic fertilizers replacing chemical fertilizers on microbially mediated rhizosphere and bulk soil processes, as well as the relationships among microbial diversity, soil properties, enzyme activity, and their effects on the quality of important medicinal herbs. *Glycyrrhiza uralensis* is one of the most extensively used traditional Chinese medicinal herbs and is also among the most widely used medicinal and food homologous species, earning it the reputation of “no herbal formula without licorice” (Egamberdieva et al, 2017). It plays a fundamental role in ensuring public health and promoting the development of the health industry (Ceccuzzi et al., 2023). Compounds like Glycyrrhizic acid, Glycyrrhizin, and Isoliquiritin as the primary pharmacological components of licorice exhibit anti-inflammatory, anti-ulcer, immune-modulatory, and synergistic effects with other drugs, playing crucial roles in clinical use, pharmacological evaluation, and quality assessment (Tao et al., 2012; Xiao et al., 2024). Therefore, studying the short-term effects of organic fertilizers replacing chemical fertilizers on the quality formation and productivity of licorice from the perspective of licorice-soil microorganisms-soil physicochemical interactions holds significant importance.

In many past studies, the close relationship between soil characteristics and soil microbiota in other ecosystems has been well established, but the focus has largely been on productivity, with minimal attention to crop flavor or medicinal herb quality (Ren et al., 2021). In this study, we hypothesize that partially substituting chemical fertilizers with organic fertilizers can greatly impact the quality formation of medicinal plants by improving soil properties, microbial diversity, and enzyme activity, thus mediating carbon-nitrogen cycling for long-term promotion of green development and enhanced nutrient effectiveness. Therefore, the objectives of this research are (1) to investigate the effects of partially substituting chemical fertilizers with organic fertilizers on the accumulation of medicinal components, yield, soil physicochemical properties, microbial diversity, and enzyme activity in licorice through metagenomic studies; (2) to explore the relationship between soil properties, enzyme activity, microbial communities, and licorice quality using interaction models. Based on these objectives, the study seeks to understand the scientific significance of rational fertilization for the formation of medicinal plant quality and the sustainable green development of soil nutrients.

## 2 Materials and methods

### 2.1 Study site

The experiment was conducted from April 2022 to November 2023 in an open field in Guyu Yuan, Yukou Town, Pinggu District, Beijing (117°12’ E, 40°07’ N), located in the northeastern plains of China. The annual average precipitation was 57.21 mm, with rainfall concentrated from July to September. The annual average temperature was 18.3°C, and the annual sunshine hours were 2579.1 h. The soil in the study area is classified as a calcareous brown soil sub-group of fire brown-yellow soil, with the dominant soil type being loessial soil. It should be noted that the experimental field was newly established by leveling previously uncultivated hilly land, and no crops had been planted in this area before the start of the study. **Table 1** summarizes the basic characteristics of the topsoil layer (0-20 cm).

**Table 1:** Basic characteristics of the topsoil layer (0–20 cm).

Soil basic properties	
pH	8.5
Total nitrogen (TN, g·kg^−1^)	0.98
Total phosphorus (TP, g·kg^−1^)	0.46
Total potassium (TK, g·kg^−1^)	29.12
Total carbon (TC, g·kg^−1^)	27.61
Soil organic matter (SOM, g·kg^−1^)	14.5

### 2.2 Experimental design

The tested licorice was a locally common variety provided by Inner Mongolia Hangjinqi Yihong Breeding Cooperative, identified by researcher Zhang Yan of the China Academy of Chinese Medical Sciences as one-year-old seedlings of the leguminous plant *Glycyrrhiza uralensis* Fisch. Licorice with approximately 6 cm of stem and approximately 20 cm of main root was selected and then transplanted into subplots.

The field experiment was conducted using a completely randomized block design, replicated three times. The experiment referenced soil nutrient content in experimental plots, field management conditions, and traditionally applied licorice fertilization rates. It involved different gradients of organic and inorganic fertilizer combinations, namely: (1) OF100: organic fertilizer [100%], (2) OF75: organic fertilizer [75%] + chemical fertilizer [25%], (3) OF50: organic fertilizer [50%] + chemical fertilizer [50%], (4) OF25: organic fertilizer [25%] + chemical fertilizer [75%], (5) OF0: chemical fertilizer [100%], and a blank control (CK) treatment. Market-available chemical fertilizers (urea [46% N], calcium superphosphate [16% P_2_O_5_], and potassium oxide [60% K_2_O]) were used (sourced from China Chemical Fertilizer Co., Ltd.), along with organic fertilizer consisting of sheep manure with an organic matter content of 458 g·kg^-1^, total nitrogen of 15.80 g·kg^-1^, total phosphorus of 17.78 g·kg^-1^, total potassium of 14.55 g·kg^-1^, alkali hydrolyzable nitrogen of 2.74 g·kg^-1^, available phosphorus of 1.45 g·kg^-1^, available potassium of 1.55 g·kg^-1^, and the pH of 7.03. The organic fertilizer was locally sourced sheep manure, processed through composting and fermentation. Fertilization rates for pure chemical fertilizer treatments were applied at 225 kg·hm^-2^ of pure N, P_2_O_5_, and K_2_O, while pure sheep manure organic fertilizer treatments were applied at 45000 kg·hm^-2^, with fertilizer quantities adjusted proportionally. The experiment comprised 18 plots, each with an area of approximately 4 m^2^ (2 m×2 m, with a ditch spacing of 20 cm), transplanting approximately 50 licorice plants per plot, surrounded by a 50 cm protective row, with row spacing of 40 cm and plant spacing of 10 cm. Although each treatment subplot measured 2 m × 2 m, five parallel rows and a three‐block randomized complete block design were implemented to ensure adequate replication and statistical power. This plot configuration aligns with standard practices in early‐stage *Glycyrrhiza uralensis* cultivation (Hou et al., 2018). The field trial selected newly reclaimed land, where surface debris was cleared two weeks before transplanting, and pre-weighed fertilizer was uniformly spread over plot beds, mixed in, and lightly covered with ditch soil. Field management followed standard licorice cultivation practices.

### 2.3 Soli and plant sampling

The licorice plants were harvested on November 23, 2023. During the harvest, licorice plants, field soil, and rhizosphere soil samples were collected from each experimental plot. Harvesting was conducted manually by uprooting whole plants to avoid mechanical damage. Loosely attached soil was gently shaken off from the roots, while tightly adhered soil was brushed off and collected as rhizosphere soil. An S-shaped sampling pattern was employed, and 15 licorice plants were sampled from each plot. Rhizosphere soil was collected by brushing the root surfaces, then sieved through a 2 mm mesh to remove debris. At the same time, bulk soil samples were collected from a depth of 10–20 cm around the root zone. To prevent cross-contamination, a sterile cotton ball was used to clean the stainless-steel shovel before each sample collection. The 15 soil samples per plot were mixed to form one composite sample, and each treatment had three biological replicates. The collected soil samples were divided into two portions: one portion was immediately placed on dry ice, flash-frozen in liquid nitrogen for 5 minutes upon arrival at the laboratory, and stored at -80°C for metagenomic sequencing. The other portion was air-dried at room temperature for physicochemical properties and enzyme activity analysis. Plant samples were processed by cleaning the roots with water to remove residual soil, then air-dried in a well-ventilated environment. These dried licorice samples were used for agronomic trait measurement and active ingredient content determination.

### 2.4 Determination of agronomic traits and active ingredient content

Agronomic traits determination: Root weight, root length, diameter at 1 cm (D1), and diameter at 20 cm (D20) of licorice samples were measured using a vernier caliper and tape measure, and the yield of medicinal parts per m^2^ was calculated. D1 and D20 represent the root thickness of the licorice and reflect overall uniformity.

Determination of active ingredient content: Seven active ingredients, namely Rutin, Liquiritin, Isoliquiritin, Liquiritigenin, Isoliquiritigenin, Glycyrrhizic acid, and Glycyrrhetinic acid A, were selected as measurement indicators based on previous studies within the research group. UPLC was employed for content determination using a Waters Symmetry C18 column (4.6 mm × 250 mm, 5 μm); the mobile phase consisted of water (0.1% formic acid) (A) and acetonitrile (B); gradient elution: 0-1.5 min, 20%-30% B; 1.5-3 min, 30%-35% B; 3-6 min, 35%-50% B; 6-8 min, 50%-65% B; 8-8.5 min, 65%-20% B; 8.5-10 min, 20%-20% B; detection wavelength at 254 nm, flow rate of 0.3 mL/min, column temperature of 40 °C, injection volume of 1 μL. Sample solution preparation: Accurately weigh 0.1 g of sample powder (passed through a 60-mesh sieve), place it in a 10 mL centrifuge tube, add 70% methanol-water solution to make up the volume, ultrasonicate (frequency 45 kHz, power 300 W) for 45 min, cool down, re-weigh, make up for the lost mass with 70% methanol-water solution, shake well, centrifuge at 12,000 rpm for 10 min, filter the supernatant through a 0.22 μm microfiltration membrane, and use the filtered solution as the sample solution. The research group has conducted methodological investigations on chromatographic conditions, and the linear relationship, precision, stability, repeatability, and average sample recovery rate all meet the requirements for quantitative analysis.

### 2.5 Soil properties and microbial enzymatic activities

Soil pH was determined using a pH meter (250A/610, Fisher Scientific, Pittsburgh, USA) with a soil: water ratio of 1:5. Electrical conductivity (EC) was measured following the method by De Vos et al. (2005). Total nitrogen (TN) content was analyzed using the Kjeldahl method (KDN-102C, Shanghai, China), and total phosphorus (TP) was quantified spectrophotometrically (UV-2550, Shimadzu, Kyoto, Japan) using molybdenum blue colorimetric analysis as described by Gong et al. (2019). Total potassium (TK) was extracted with Na_2_CO_3_ and quantified using flame photometry (FP6400A, Shanghai, China) as previously reported (Wu et al., 2021). Total carbon (TC) content was determined through combustion oxidation and analyzed using a carbon analyzer (Multi N/C 3100, German). Soil organic carbon (SOC) and soil organic matter (SOM) were determined post K_2_Cr_2_O_7_ oxidation and FeSO_4_ titration following the method by Akhtar et al. (2018). Available potassium (AK) and available nitrogen (AN) were assessed by atomic absorption and alkaline hydrolysis diffusion methods, respectively (Chen et al., 2018).

Soil enzyme activities were determined according to the instructions of the Solarbio assay kits. Soil urease (S-UE) activity was measured using the indophenol blue colorimetric method, with absorbance read at 630 nm using a visible spectrophotometer. Soil sucrase (S-SC) activity was determined using the 3,5-dinitrosalicylic acid colorimetric method, with absorbance measured at 540 nm. Soil alkaline phosphatase (S-AKP) activity was assessed using the borate buffer colorimetric method, and absorbance was recorded at 660 nm. Soil alkaline phosphatase (S-ALPT) activity was determined using the copper salt colorimetric method, with absorbance measured at 680 nm. Soil cellobiohydrolase (S-CBH) activity was measured using the p-nitrophenol colorimetric method, with absorbance read at 405 nm. Detailed procedures can be found in the corresponding Solarbio assay kit manual (www.solarbio.com).

### 2.6 DNA extraction, metagenomics sequencing, and bioinformatic analysis

Total soil DNA was extracted from 0.5 g of mixed fresh soil using the FastDNA^®^ SPIN Kit for Soil (MP Biomedicals, USA) according to the manufacturer’s instructions. The quality and concentration of soil DNA were measured using a NanoDrop 2000 spectrophotometer (Thermo Scientific, USA), and samples were then subjected to metagenomics sequencing. Approximately 1 μg of DNA from each sample was sent to LingEn Biotechnology Co., Ltd. (Shanghai, China), and analyzed with an Illumina HiSeq 4000 platform (Illumina Inc., San Diego, CA, USA) using a HiSeq 3000/4000 PE Cluster and 3000/4000 SBS Kits.

The bacterial, fungal, and viral communities in the rhizosphere and bulk soil samples of licorice were characterized through metagenomic sequencing, yielding 36 sets of raw data. Subsequently, the raw metagenomic sequences were subjected to quality control using Fastp ver. 0.20.0 (https://github.com/OpenGene/fastp) to remove adapters, reads shorter than 50 bp with an average quality score below 20, and those containing N bases. The clean reads were assembled into contigs using Megahit ver. 1.1.2 (http://www.l3-bioinfo.com/products/megahit.html) with optimized k-mer parameters. Contigs longer than 300 bp were used to predict open reading frames (ORFs) using MetaGene (http://metagene.cb.k.u-tokyo.ac.jp/), which were then translated into amino acid sequences. All predicted genes were clustered into a non-redundant gene catalog using CD-HIT ver. 4.6.1 (http://www.bioinformatics.org/cd-hit/) with 95% similarity and 90% coverage thresholds. Clean reads from each sample were mapped to the non-redundant gene catalog generated in the previous step using SOAPaligner ver. 2.2.1 (http://soap.genomics.org.cn/soapaligner.html), and the number of reads corresponding to functional genes in each sample was calculated. Functional annotation of the non-redundant gene catalog was performed using Diamond ver. 0.8.35 (https://github.com/bbuchfink/diamond).

### 2.7 Statistical analysis

The normality and homogeneity of variance of the data were assessed using the Shapiro–Wilk test and Levene’s test, respectively. For datasets that did not follow a normal distribution, a natural logarithm transformation was applied to meet the assumptions of parametric tests. All statistical analyses were conducted using SPSS 24.0 software, and results were expressed as the mean ± standard deviation (SD). Before performing one-way analysis of variance (ANOVA), data normality was confirmed. Differences among treatments were evaluated using Duncan’s Multiple Range Test (DMRT) at a significance level of *p* ≤ 0.05. For microbial community analysis, alpha diversity was assessed using the Shannon and Chao1 indices, while beta diversity was evaluated through Principal Coordinate Analysis (PCoA). Microbial taxa with a relative abundance greater than 1.0% in the rhizosphere were included in community structure analysis. Analysis of Similarities (ANOSIM) with 999 permutations was used to test differences in beta diversity. To explore the relationships between soil microbial communities and environmental factors, Redundancy Analysis (RDA) was performed using Canoco software. Spearman correlation analysis was applied to examine associations among soil microbial diversity, enzyme activity, and physicochemical properties. In addition, Partial Least Squares Path Modeling (PLS-PM) was used to investigate the interactions among soil physicochemical characteristics, microbial communities, soil enzyme activities, and the accumulation of plant active ingredients. Mantel tests, implemented in R software (v4.2.2; http://www.r-project.org/), were conducted to evaluate correlations between metagenomic profiles, soil properties, and compound contents. Bar charts depicting microbial species composition were generated using GraphPad Prism 8, while all other figures were prepared using Origin 2021 and Adobe Illustrator CC 2021 for improved clarity and visual presentation.

## 3 Result

### 3.1 Growth and development of licorice and accumulation of active ingredients

The research shows that varying fertilization levels significantly affect licorice growth and development (**Supplementary Table S1**). As chemical fertilizer usage increases, licorice exhibits an initial growth phase followed by a decline in root length, weight, D1, D20, and yield. Notably, root weight and yield are notably higher under each fertilization treatment compared to the control group. Pure organic (OF100) and pure chemical (OF0) fertilization are notably less effective than combined applications.

**Figure 1:**
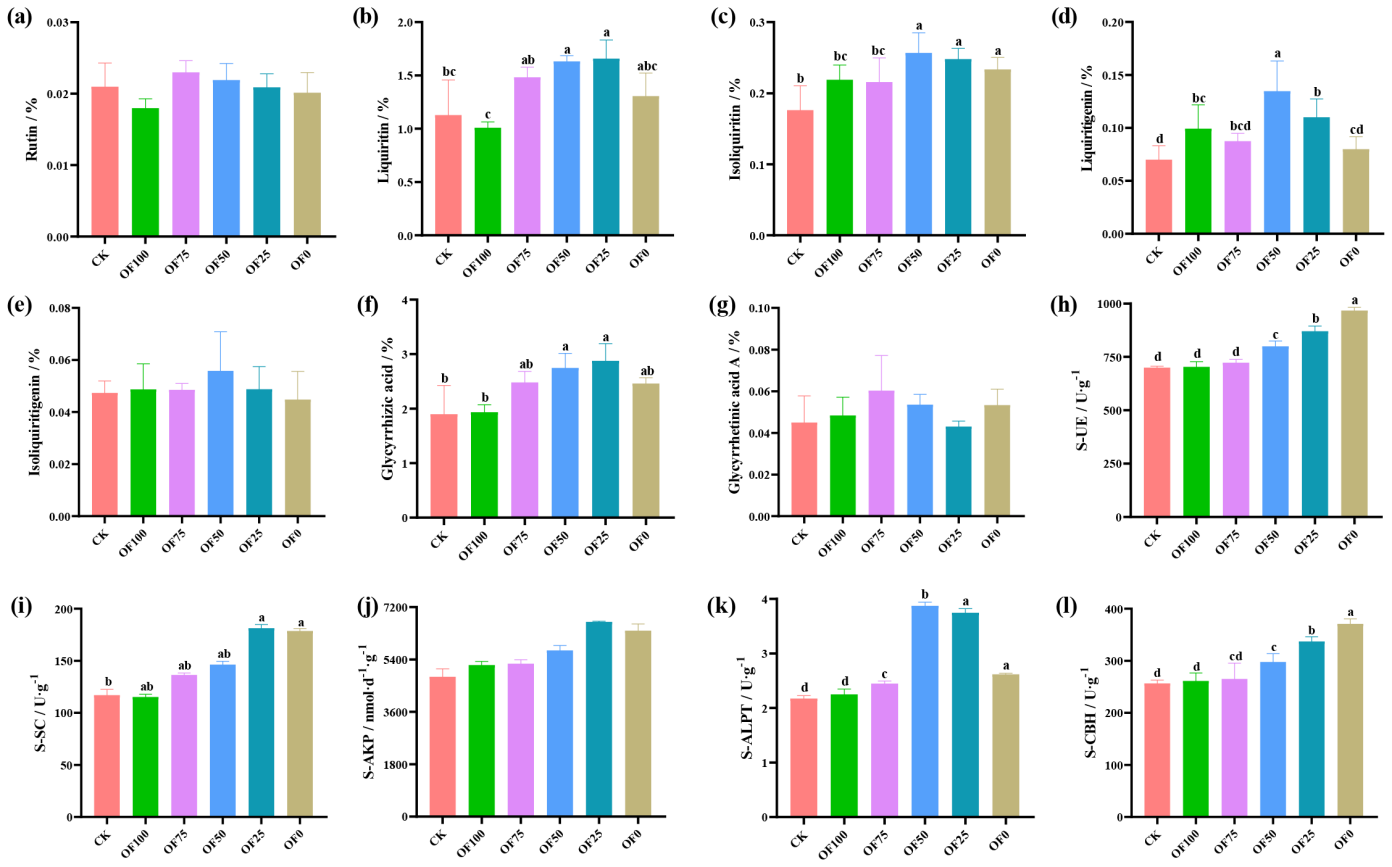
Comparison of active ingredient contents **(a-g)** and soil enzyme activities **(h-l)** of licorice under different fertilization treatments. Different lowercase letters within the same panel indicate statistically significant differences between treatments at *p* ≤ 0.05, as determined by Duncan’s Multiple Range Test (DMRT). Treatments sharing the same lowercase letter are not significantly different from each other.

Compared with the CK group, optimized fertilizer combinations significantly affect the accumulation of key compounds in licorice, including Glycyrrhizin, Isoliquiritigenin, Glycyrrhetinic acid, and Glycyrrhizic acid, while Liquiritin, Isoliquiritigenin, and Glycyrrhizic acid A show no significant differences (**Figures 1a-g**). With increased chemical fertilizer proportions, licorice root content of seven effective components initially rises and then declines. Specifically, in OF25, Glycyrrhizin, and Glycyrrhetinic acid content peaks, increasing by 47.1% and 51.8% respectively compared to CK (*p* ≤ 0.05). In OF50, Isoliquiritigenin, Glycyrrhetinic acid, and Isoliquiritigenin content peaks, increasing by 45.7%, 92.4%, and 17.9% respectively compared to CK. Overall, pure organic or chemical fertilizers have an insignificant impact on licorice’s effective components, especially Glycyrrhizin and Glycyrrhizic acid, where OF50 and OF25 perform best.

### 3.2 Soil physicochemical properties and enzymatic activities

Organic fertilizers partially replacing chemical counterparts significantly influence soil physicochemical properties (**Supplementary Table S2**). Soil pH ranges from 7.94 to 8.51 across treatments, notably decreasing with higher chemical fertilizer proportions. OF100 shows peak levels of AK, AN, TP, TC, SOM, and SOC, significantly different from CK (*p* ≤ 0.05). Conversely, OF50 records the lowest levels of AN, TK, TN, TP, TC, and EC, while AK, OM, and SOC peak in OF0. Notably, EC is highest in OF0, indicating increased ion content due to pure chemical fertilization. Except for pH and EC, other soil nutrient indices decline consistently with increasing chemical fertilization, notably, AK increasing by 75.0% and 80.5% compared to CK and OF0, respectively.

Analyzing soil enzyme dynamics under different fertilization schemes (**Figures 1h-l**) reveals nuanced patterns. S-UE and S-CBH peak in OF0, significantly different from CK (*p* ≤ 0.05), increasing with higher chemical fertilization. Conversely, S-SC and S-AKP peak in OF25, while S-ALPT peaks in OF50, all significantly different from other groups (*p* ≤ 0.05). These enzyme dynamics closely correlate with carbon, nitrogen, and phosphorus transformations. Notably, enzyme activity increases across all treatments compared to CK and pure organic fertilization, highlighting the complex soil-enzyme interplay under diverse fertilization strategies.

### 3.3 Combined analysis of fertilization treatments on licorice quality and soil quality

**Figure 2:**
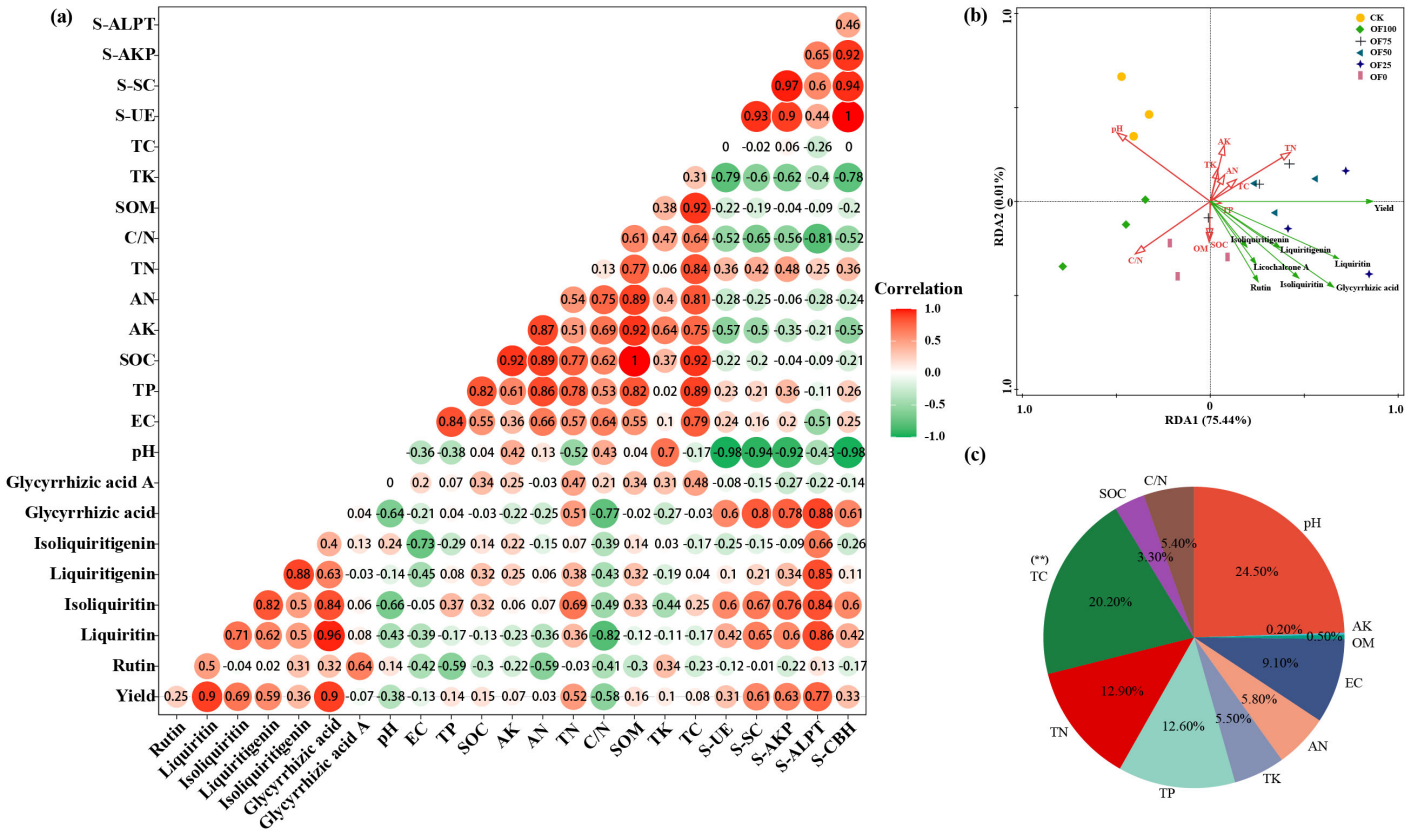
Bidirectional correlation analysis among various indices under different fertilization treatments **(a)**, RDA analysis between soil properties and licorice yield and quality **(b)**, and contribution rates **(c)**. Colors shifting towards red indicate stronger positive correlations, while those shifting towards green indicate stronger negative correlations.

Correlation analysis revealed a significant positive link (*p* ≤ 0.01) between Glycyrrhizin, Glycyrrhetinic acid, and yield (**Figure 2a**), suggesting that optimal fertilization can enhance both licorice yield and quality simultaneously. Additionally, a notable negative correlation (*p* ≤ 0.05) was found between Glycyrrhizin, Glycyrrhetinic acid, and the C/N ratio, with a weak negative trend observed in soil physicochemical properties, except for a slight positive correlation with TN. Soil enzyme activity showed a significant positive connection (*p* ≤ 0.05) with Glycyrrhetinic acid, Glycyrrhizin, Glycyrrhizic acid, and Isoliquiritigenin, particularly with protease, while exhibiting weaker positive links with other enzyme activities. Moreover, pH exhibited a highly significant negative correlation (*p* ≤ 0.01) with all enzyme activities except protease. The RDA plot (**Figures 2b, c**) indicated that licorice yield correlated positively with TP, TN, TC, AN, AK, and TK, but negatively with pH and C/N. Conversely, the seven active components, including Glycyrrhetinic acid and Glycyrrhizin, showed positive links with OM, SOC, TP, and TN, with TN showing the strongest correlation, and negative correlations with pH and C/N, aligning with the earlier correlation analysis. Hence, the yield and quality formation of licorice is positively related to the ratio of N/C and enzyme activity, while it is weakly related to elements such as P and K.

Using the CRITIC method, we analyzed the weights of eight indicators, including yield and active components (Glycyrrhetinic acid, Glycyrrhizin, etc.), under different fertilization treatments (**Supplementary Tables S3, S4**). For medicinal quality and yield, the weights ranked as follows: Glycyrrhetinic acid, Liquiritin, Isoliquiritigenin, yield, Isoliquiritin, Glycyrrhizic acid, and Glycyrrhizin. Notably, stronger correlations between conflicting indicators resulted in lower conflict and smaller weights. CRITIC comprehensive scoring evaluated medicinal material performance in various fertilization treatment groups, with OF50, OF25, and OF75 ranking highest and the CK group lowest.

### 3.4 Soil microbial diversity and composition

Metagenomic sequencing was conducted on bulk (BOF group) and rhizosphere (ROF group) soils from six treatment groups, totaling 36 samples. After quality control and filtering, average clean reads and contigs were 36,408,922 and 23,724, respectively. Soil bacteria and fungi α-diversity (phylum level) were assessed using Chao1 and Shannon indices. We found that rhizosphere microorganisms were consistently more abundant and diverse than bulk soil (**Supplementary Figures S1A-D**). Bacterial diversity was generally higher in the organic-chemical fertilizer groups compared to CK, notably with ROF75 showing significant differences (*p* ≤ 0.05). Single fertilizer impacts were relatively stable or decreasing. Fungal diversity increased significantly with OF75 in bulk soil but decreased in the rhizosphere, opposite for OF25. NMDS analysis (**Supplementary Figures S1E-H**) showed significant influences of fertilizer gradients on soil fungal β-diversity in both soils (*p* ≤ 0.05). Soil bacterial β-diversity was similar between CK and combined fertilizers, influenced by OF0 and OF100 treatments.

**Figure 3:**
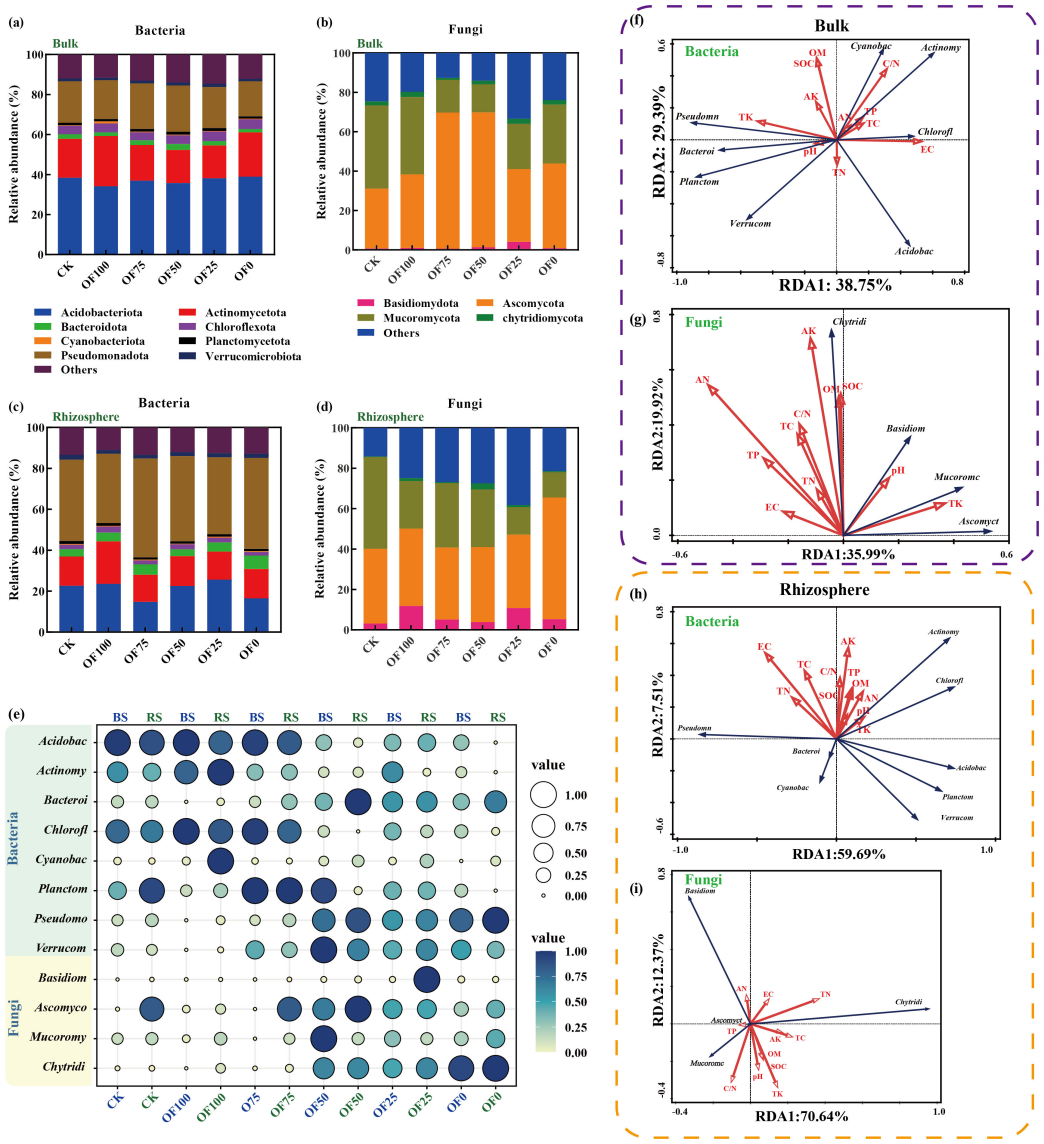
**(a–d)** Composition of bacterial and fungal communities in bulk soil **(a, b)** and rhizosphere soil **(c, d)** under different treatments. **(e)** Bubble plot comparing relative abundances of major microbial taxa across treatments. Darker colors represent higher species richness within a treatment, and circle sizes correspond to relative abundance. **(f–i)** Redundancy Analysis (RDA) illustrating relationships between soil physicochemical properties and dominant bacterial species. Bulk' and 'BS' refer to microbial diversity in bulk soil, while 'Rhizosphere' and 'RS' refer to microbial diversity in rhizosphere soil. Abbreviations of microbial taxa: Acidobac, Acidobacteriota; Actinomy, Actinomycetota; Bacteroi, Bacteroidota; Chlorofl, Chloroflexota; Cyanobac, Cyanobacteriota; Planctom, Planctomycetota; Pseudomo, Pseudomonadota; Verrucom, Verrucomicrobiota; Basidiom, Basidiomycota; Ascomyco, Ascomycota; Mucoromy, Mucoromycota; Chytridi, Chytridiomycota.

Different organic fertilizer regimes significantly altered bacteria community composition in both bulk and rhizosphere soils at various taxonomic levels (**Figures 3a, c, e**; **Supplementary Tables S5**, **S6**). Actinobacteria increased notably in BOF100 but decreased in BOF25 and BOF50; conversely, Bacilli and Verrucomicrobia increased significantly. Rhizosphere bacteria saw increased Actinobacteria, Chloroflexi, and Pseudomonadales in ROF100 and ROF75, with a decrease in Acidobacteria. Cyanobacteria and Sordariomycetes rose in OF100, while Anaerolineae decreased; Acidimicrobiia increased in organic fertilizer replacement. At the order level, differences between rhizosphere and bulk soil bacteria were more pronounced, especially in Rhizobiales, Acidobacteriales, Solirubrobacterales, Streptomycetes, Rhodospirillales, Anaerolineales, and Myxococcales in ROF50 and ROF100, with Acidobacteriales showing significant increases across all treatments. As for fungi, the dominant phyla were Ascomycota, Basidiomycota, Zygomycota, and Glomeromycota (**Figures 3b, d, e**; **Supplementary Tables S7**, **S8**). At the phylum level, organic fertilizer replacement boosted Ascomycota and Basidiomycota in bulk soil, while Basidiomycota dominated in the rhizosphere without organic fertilizer. Orders Agaricales and Agaricomycetales prevailed in all fertilized groups, with Orbiliales decreasing notably in replacement groups.

### 3.5 Correlation analysis of microbial-licorice-soil

**Figure 4:**
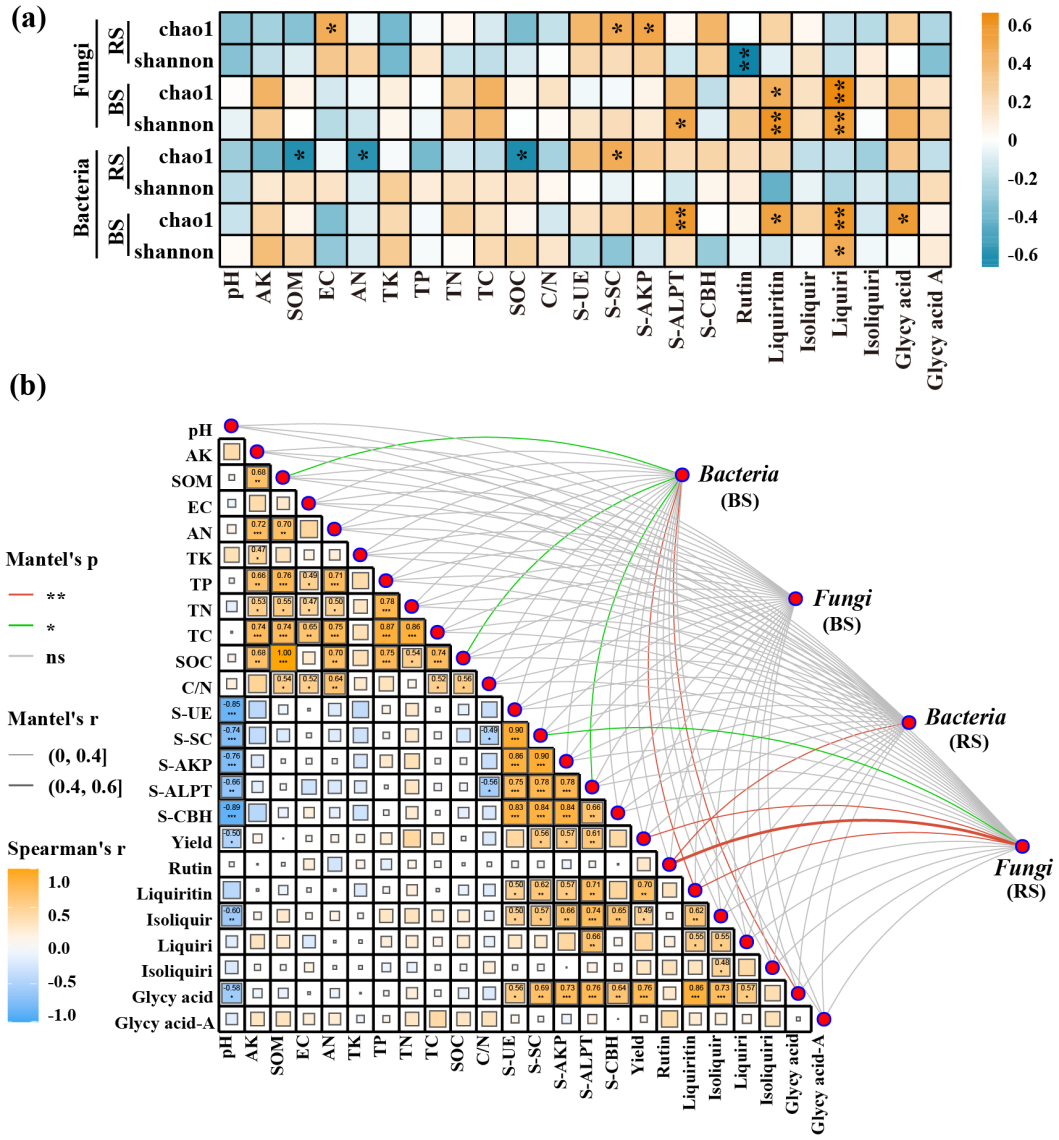
Correlation analysis between soil physicochemical properties, enzyme activities, and microbial communities at the genus level **(a)**, and Mantel test results **(b)**. The asterisk (*) indicates a significant difference between two indicators (p ≤ 0.05), while the double asterisks (**) indicate a highly significant difference between two indicators (p ≤ 0.01). Green lines represent statistically significant correlations (*p* ≤ 0.05), while red lines indicate highly significant correlations (*p* ≤ 0.01) between soil/environmental indicators and bacterial or fungal diversity. Correlation strength is indicated by the r-value, with *p* ≤ 0.05 considered meaningful. All correlation analyses were performed using Spearman’s rank correlation. BS, Bulk Soil; RS, Rhizosphere Soil; Isoliquir, Isoliquiritin; Liquiri, Liquiritigenin; Isoliquiri, Isoliquiritigenin; Glycy acid, Glycyrrhizic acid; Glycy acid A, Glycyrrhetinic acid A.

RDA analysis and mantel test coefficients revealed the correlations at the phylum level between soil physicochemical properties and the composition of microbial communities in both the bulk and rhizosphere soils. There were significant differences in soil microbial diversity between the bulk and rhizosphere soils shaped by soil physicochemical properties. Specifically, in the bulk soil, the abundance of Actinobacteria was positively correlated with TP, TC, AN, and C/N, while negatively correlated with pH and TK. Conversely, in the rhizosphere soil, the abundance of Actinobacteria showed positive correlations with pH, TK, and OM. Similarly, in the fungal community, the distribution of Zygomycota in bulk soil showed positive correlations with soil TN and pH levels, while negatively correlated with the abundance of Zygomycota in the rhizosphere (**Figures 3f-l**). In the Mantel test, it was found that only SOM and SOM-related soil physicochemical properties exhibited significant differences (*p* ≤ 0.05) in microbial community composition in the bulk soil. Accumulation of effective components such as glycyrrhizin and glycyrrhetinic acid seemed closely associated with fungal composition in the rhizosphere soil and bacterial composition in the bulk soil, showing a tighter positive correlation with bacterial and fungal α-diversity in the bulk soil (**Figures 4A, B**).

RDA analysis and Mantel test coefficients revealed the correlations at the phylum level between microbial community composition and soil enzyme activities. In the bulk soil, the phylum Proteobacteria showed a positive correlation with S-ALPT activity and a negative correlation with S-UE, S-CBH, and S-SC activities. Interestingly, this trend was significantly diverged in the rhizosphere bacterial community. For the fungal community, it was found that fungi, especially Basidiomycota, in the rhizosphere showed close positive correlations with various soil enzyme activities, while in the bulk soil, only S-ALPT showed some correlation with Basidiomycota and Zygomycota. The Mantel test results demonstrated a strong correlation between S-ALPT and the bulk bacterial community and between S-SC and the rhizosphere fungal community (**Supplementary Figure S2**).

### 3.6 PLS-PM equation modeling and co-occurrence network

Random forest analysis indicates that soil enzyme activity, pH, fungal composition, and total TC are key variables influencing the synthesis of important metabolites such as Glycyrrhizic acid and Liquiritin. PLS-PM analysis reveals that substituting organic fertilizers for some portion of chemical fertilizers directly influences soil enzyme activity and the microbial communities of both bulk and rhizosphere soils through positive feedback mechanisms, while indirectly affecting soil physicochemical properties, particularly SOC and TC content. This, in turn, modulates the synthesis of secondary metabolites in licorice plants (**Supplementary Figure S3**).

The co-occurrence network revealed shifts in ecological patterns in licorice’s bulk and rhizosphere soil microbial communities post various fertilization treatments. Bacteria communities showed greater size and interconnectedness compared to fungi, with modular family structures across all six treatment groups (**Supplementary Table S9**). Surprisingly, in this network, except for pure chemical fertilizer, all other fertilization methods saw notable reductions in node and edge numbers, average degree, and density compared to the CK control group. This indicates simplified microbial network complexity under combined fertilization, reducing information, energy, and nutrient exchanges between communities and microbe interactions. Conversely, pure chemical fertilizer intensified interaction networks among key microbial species and enhanced their centrality (**Supplementary Figure S4**).

## 4 Discussion

### 4.1 Effect of different fertilization regimes on quality of licorice and soil fertility

Proper fertilization plays a crucial role in enhancing the quality and increasing the yield of medicinal plants. The combined application of organic and chemical fertilizers not only ensures a rapid nutrient supply but also provides long-lasting nutrition to plants, thus stably supporting their growth and metabolism (Ren et al., 2021; Qiao et al., 2019; Lazcano et al., 2013). This study reveals that within the range of different ratios of organic to chemical fertilizers (OF25-OF75), compared to using only CK or a single fertilizer, there is a significant increase in the yield of medicinal parts of licorice, along with an increase in the content of active compounds such as Glycyrrhizic acid, Glycyrrhizin, and Isoliquiritin (**Figure 1**). This phenomenon can be attributed to a combination of factors: firstly, plants obtain a more comprehensive nutrient supply, leading to improvements in soil structure, increased soil permeability, and water retention capacity; secondly, organic matter provides energy to soil microorganisms, promoting their reproductive activities (Chomel et al., 2016). Building upon these findings, this study further investigates the impact mechanisms of soil physicochemical properties and microorganisms on the formation of licorice quality, highlighting the importance of soil-microbe-plant interactions.

Soil nutrients serve as the elemental basis for the synthesis of primary and secondary metabolites in medicinal plants. Meanwhile, soil physicochemical properties are considered indicators of soil quality, defined as the ability of soil to maintain environmental quality and enhance plant productivity (Oladele, 2019). The study finds that the addition of organic fertilizer improves the content of key physicochemical indicators such as AK, TN, SOM, TC, etc., enhancing both soil fertility and nutrient supply within a certain range of organic fertilizer ratios. This enhancement is evidently due to the impact of organic fertilizer application on soil, resulting in higher humic acid concentrations, dilution of soil aeration, and denser soil mineral composition, which may lead to greater porosity and structural stability in soil, thereby further improving root-zone nutrient supply (Mao et al., 2015). The CRITIC weighting method results indicate that the comprehensive score of medicinal materials under the OF50 treatment ranks first, while OF0 and OF100 rank 4th and 5th, respectively, highlighting the necessity of proper fertilization for the quality formation of medicinal materials and the utilization and management of soil nutrients while considering ecological benefits. The improvement in soil conditions stems from the supply of soil organic matter substrates and mineralized nutrients, ultimately enhancing plant productivity (Liu et al., 2021). However, it is regrettable that this study only monitored for eight months, and given the long-lasting and gradual nature of organic fertilizers, the patterns of short-term changes in soil physicochemical properties may vary with the duration of application, thus requiring further observation.

### 4.2 Effects of different fertilization regimes on bulk and rhizosphere microbial shaping

Generally, fertilization management systems impact soil properties and microbial diversity, with soil microbes playing various ecological roles, such as nutrient cycling through crop residue decomposition and promoting crop growth (Rodriguez-Berbel et al., 2020). While past notions focused on rhizosphere processes determining nutrient utilization and emphasized the dominance of chemical fertilizers (Hartmann et al., 2008; Ai et al., 2012), the reality includes the direct influence of microbial changes in nearby bulk soil on rhizosphere communities. A comprehensive understanding of microbially mediated rhizosphere processes, their relations with soil physicochemical properties and microbial communities, and their association with soil enzyme activities may help identify key drivers of soil nutrient effectiveness, potentially enhancing crop growth. Our findings indicate that organic fertilizer substitution increases the α-diversity of bacteria and fungi, yet their performance differs significantly between bulk and rhizosphere areas, affirming changes in microbial community structure in response to nutrient environmental resource differences due to ecological niche variations. Moreover, based on response analysis of soil community structure to organic fertilizer substitution for chemical fertilizers, we confirm a significant impact on soil microbial β-diversity (**Supplementary Figure S1C-D**). Mantel tests reveal a significant correlation between SOC and SOM with bacterial relative species abundance, while their correlation with fungi seems less pronounced (**Figure 4**). Previous studies suggest that bacteria are generally more sensitive to C substrates than fungi due to their shorter turnover times, explaining the observed changes in bacteria in our fertilized soils (Kuzyakov, 2010).

The diversity and composition of soil microbial communities not only reflect biological conversion efficiency but also soil fertility status, with management, land use, and crop structure potentially affecting microbial community assembly processes (Manasa et al., 2020; Montiel-Rozas et al., 2018; Alvarez-Martin et al., 2016). In this study, major bacterial phyla, such as Actinobacteria and Pseudomonas, significantly increased with organic fertilizer addition, while Acidobacteria decreased. Actinobacteria are highly abundant bacteria utilizing lignocellulosic, semi-cellulosic, proteinaceous, cellulose, and other C/N substrates for rapid growth, thus requiring more carbon and nitrogen than other bacteria (Dai et al., 2018; Liu et al., 2019), further explaining their increase and close association with higher SOC and TC in organic substitution treatments. Another explanation may relate to Actinobacteria’s composition, with Actinomycetes being strictly aerobic bacteria; partial organic fertilizer substitution enhances sufficient oxygen availability and porosity, enriching conditions favorable for their growth, thus supporting the notion that appropriate organic substitution aids in improving soil properties (Tian et al., 2015). Similarly, a strong negative correlation between SOC and Acidobacteria suggests their lack of competitiveness, thus exhibiting lower abundance under organic substitution treatments (Filippidou et al., 2016). Moreover, other taxonomic groups, especially Acidobacteria, show negative responses to changes in soil SOC and TK (**Figure 3f**), possibly related to ecological strategies in these groups (Kalam et al., 2020). Regarding fungal communities, dominant phyla in organic substitution treatments include Ascomycota and Basidiomycota. Unlike bacteria, these fungal phyla show less pronounced responses to changes in soil physicochemical properties induced by organic substitution. Ascomycota primarily responds positively to TP and AN, likely due to their function as crucial decomposers in farmland (Clemmensen et al., 2013). Reported impacts on their abundance include soil organic matter content, and previous research has confirmed conditions required for Ascomycota growth are related to soil nitrogen (Jing et al., 2016; Fontaine et al., 2011), further supported by their highest abundance under OF75. Overall, soil bacterial microbial community composition is primarily influenced by SOC and SOM, while fungal composition may be closely related to nitrogen effectiveness.

Functional capabilities of soil microbial communities are reflected in enzyme activity involved in nutrient mineralization processes, serving as key indicators of soil nutrient status and microbial metabolic activity (Lin et al., 2017; Xiao et al., 2018). For instance, soil microbes produce sucrase, which decomposes sucrose to provide carbon sources and energy (Yang et al., 2024). Our study found that excessive chemical fertilizers inhibit soil sucrase, alkaline protease, and alkaline phosphatase activities, while co-application with organic fertilizers alleviates this inhibition. These enzymes are key substances involved in soil carbon, nitrogen, and phosphorus cycles, providing carbon, nitrogen, and effective phosphorus to promote microbial diversity. Meta-analysis consistently shows that co-application of organic fertilizers increases activities of urease, sucrase, and phosphatase (Burke et al., 2011), consistent with our observations of significantly increased S-SC and S-AKP activities under organic substitution treatments in this study, but opposite to decreased urease activity. Furthermore, we observed positive correlations between enzyme activities in bulk soil, except for S-ALPT, with Acidobacteria, and negative correlations with Proteobacteria; however, a significant reversal occurs in the rhizosphere (**Supplementary Figure S2**), indicating opposing responses of rhizosphere and bulk microbial communities to enzyme activity, possibly due to symbiotic or competitive relationships between rhizosphere microbes and plant root systems, influencing microbial community structure, activity, and enzyme activity manifestation (Oakley et al., 2010), necessitating further in-depth research and analysis. In our study, we confirmed a positive correlation between soil enzyme activity and secondary metabolites such as Glycyrrhizic acid and Glycyrrhizin, as well as a positive correlation with nitrogen and a negative correlation with carbon, further elucidating the extensive relationship between soil enzyme activity, soil physicochemical properties, and secondary metabolite synthesis. However, soil properties’ influence on soil enzyme activity may be indirect. Random forest and PLS-PM analyses confirm that soil physicochemical properties indirectly affect soil enzyme activity by directly influencing microbial (bacterial and fungal) community composition, ultimately affecting licorice quality formation (**Supplementary Figure S3**). Furthermore, this study focused on the first growing season of one-year-old *G. uralensis* seedlings, reflecting commercial harvesting practices in northern China, where licorice is commonly harvested after one season for medicinal use. We acknowledge that multi‐season studies could further elucidate the long-term effects of organic fertilization and recommend this as a direction for future research.

## 5 Conclusion

Organic fertilizer substitution enhances licorice yield, bioactive compound accumulation, and soil nutrient status, supporting sustainable agricultural practices. Compared to single fertilization, mixed organic amendments reduce soil pH and EC, alleviate acidification, and increase AK, TC, SOM, and SOC contents, alongside S-AKP and S-ALPT activities. Organic inputs promote bacterial α-diversity in both bulk and rhizosphere soils, while fungal α-diversity increases exclusively in the rhizosphere. Actinobacteria exhibit significant enrichment, whereas Ascomycota decline in bulk soil under organic fertilization. Variations in SOM, SOC, and AN strongly influence microbial diversity, and specific microbial taxa likely mediate S-SC, S-AKP, and S-ALPT activities while regulating glycyrrhizin, glycyrrhetinic acid, and glycyrrhizic acid biosynthesis. These findings underscore the short-term microbial responses to organic amendments and their role in shaping medicinal quality through licorice-soil-microbe interactions. Further investigations into functional gene expression and nutrient turnover processes are warranted to optimize fertilization strategies for improved soil microbial function and secondary metabolite synthesis.

## Data Availability

The original contributions presented in this study are publicly available. The datasets presented in NCBI, PRJNA1276867.

## Supplementary material

Supplementary Figure 1 Under different fertilization treatments, a-diversity **(a–d)** and b-diversity **(e–h)** of bulk and rhizosphere soils. Panels a and c represent bacterial diversity, while panels b and d represent fungal diversity. Higher Chao1 values indicate greater species richness, and higher Shannon indices indicate higher diversity. Different lowercase letters within the same group indicate significant differences (p ≤ 0.05). 'Bulk or BS' refers to microbial diversity in bulk soil, while 'Rhizosphere or RS' refers to microbial diversity in rhizosphere soil.

Supplementary Figure 2 RDA of soil enzyme activities and dominant microbial taxa. “Bulk” indicates microbial communities in bulk soil, while “rihizospher” refers to those in rhizosphere soil. Abbreviations of microbial taxa: Acido-bac, Acidobacteriota; Actinomy, Actinomycetota; Bacteroi, Bacteroidota; Chlorofl, Chloroflexota; Cyanobac, Cyanobacteriota; Planctom, Planctomycetota; Pseudomo, Pseudomonadota; Verrucom, Verrucomicrobiota; Basidiom, Basidiomycota; Ascomyco, Ascomycota; Mucoromy, Mucoromycota; Chytridi, Chytridiomycota.

Supplementary Figure 3 Random Forest model and PLS-PM equation for significant secondary metabolite accumulation in licorice under different variables. In the Random Forest model, “*” indicates variables significantly associated with the accumulation of licorice secondary metabolites. In the PLS-PM equation, red indicates positive correlation, black indicates negative correlation. The path diagram represents the correlations between each pair of variables. “**” denotes highly significant correlation (*p* ≤ 0.01), and ““ denotes significant correlation (*p* ≤ 0.05) between the variables.

Supplementary Figure 4 Analysis of the co-occurrence network of dominant bacteria and fungi. The co-occurrence network diagrams depict symbiotic patterns between bacterial and fungal species in soil samples from bulk and rhizosphere soils under different fertilization treatments. Edge colors represent positive (purple) and negative (green) correlations. Connections repre-sent strong correlations (Spearman’s r > 0.85) and significant correlations (*p* ≤ 0.01). Nodes in the co-occurrence networks are colored by phylum level.
