# Supplementary figures and images for: Mechanistic insights from metagenomics into the early-stage quality improvement of licorice under partial replacement of chemical by organic fertilizers
Source: Front Plant Sci. 2025 Jun 25;16:1613771. doi: 10.3389/fpls.2025.1613771 (PMC12239752; doi:10.3389/fpls.2025.1613771)

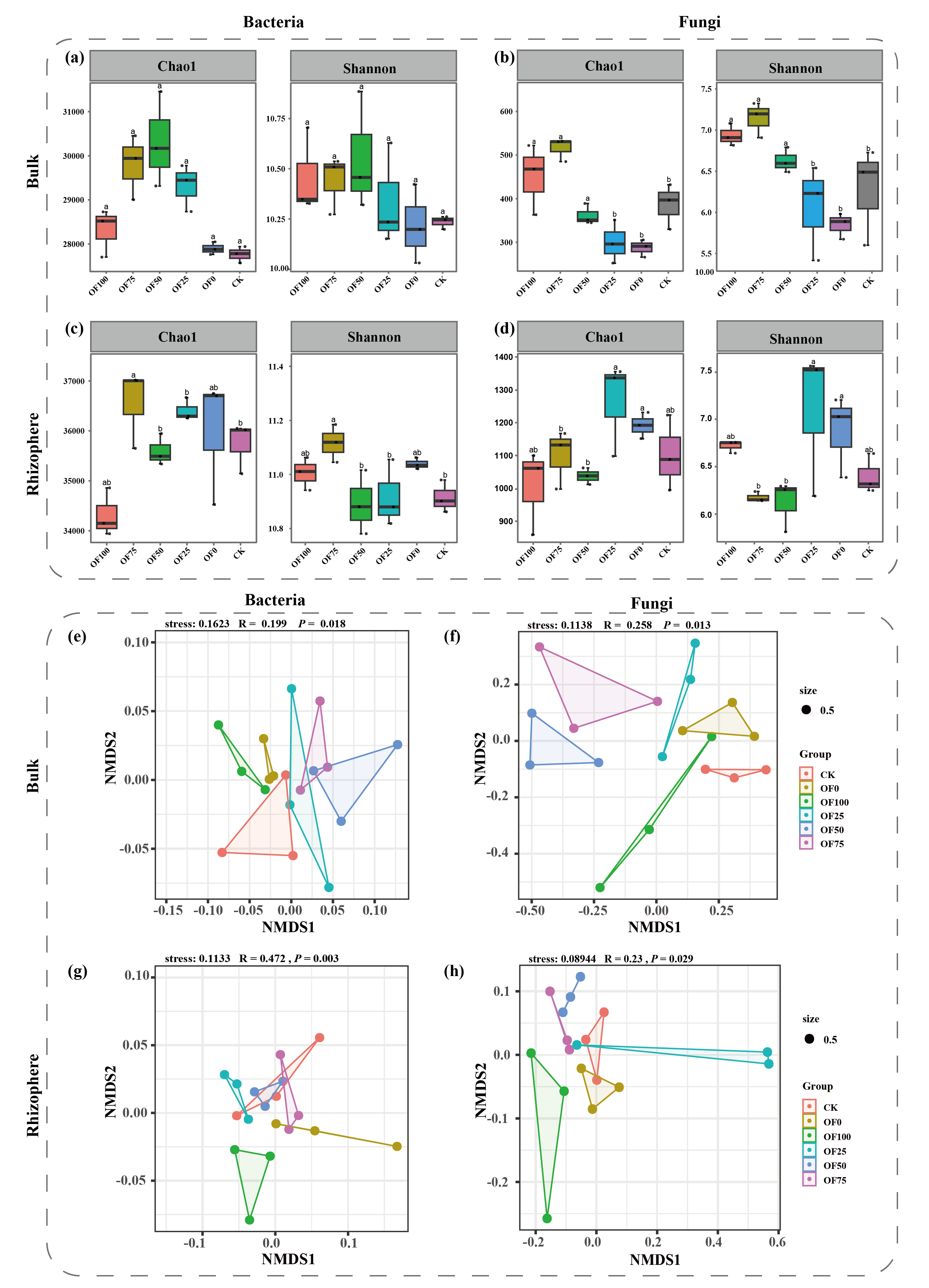

Supplement: Supplementary Figure 1 — Under different fertilization treatments, a-diversity (a–d) and b-diversity (e–h) of bulk and rhizosphere soils. Panels a and c represent bacterial diversity, while panels b and d represent fungal diversity. Higher Chao1 values indicate greater species richness, and higher Shannon indices indicate higher diversity. Different lowercase letters within the same group indicate significant differences (p ≤ 0.05). 'Bulk or BS' refers to microbial diversity in bulk soil, while 'Rhizosphere or RS' refers to microbial diversity in rhizosphere soil. [file Image1.png]

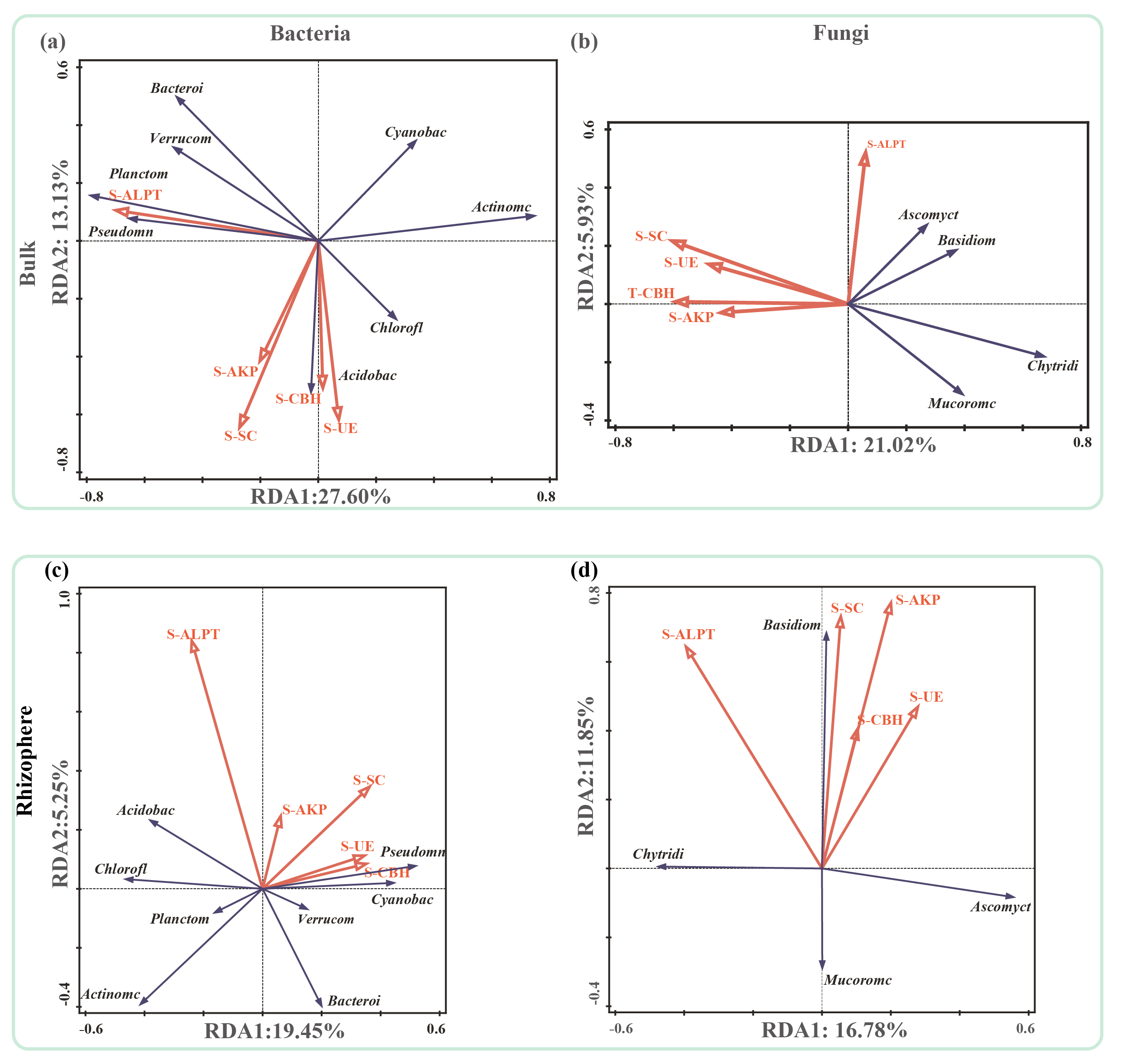

Supplement: Supplementary Figure 2 — RDA of soil enzyme activities and dominant microbial taxa. “Bulk” indicates microbial communities in bulk soil, while “rihizospher” refers to those in rhizosphere soil. Abbreviations of microbial taxa: Acido-bac, Acidobacteriota; Actinomy, Actinomycetota; Bacteroi, Bacteroidota; Chlorofl, Chloroflexota; Cyanobac, Cyanobacteriota; Planctom, Planctomycetota; Pseudomo, Pseudomonadota; Verrucom, Verrucomicrobiota; Basidiom, Basidiomycota; Ascomyco, Ascomycota; Mucoromy, Mucoromycota; Chytridi, Chytridiomycota. [file Image2.png]

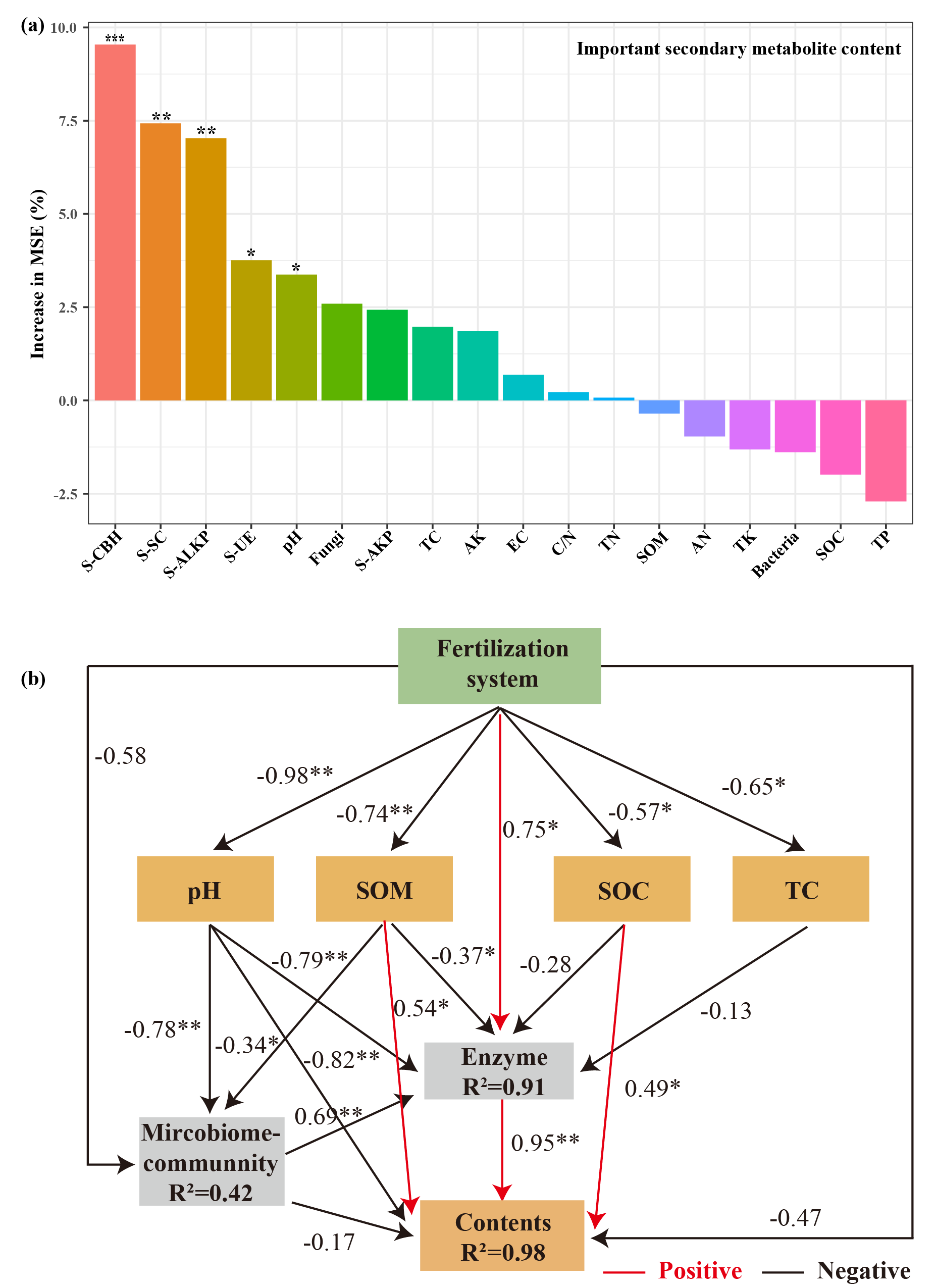

Supplement: Supplementary Figure 3 — Random Forest model and PLS-PM equation for significant secondary metabolite accumulation in licorice under different variables. In the Random Forest model, “*” indicates variables significantly associated with the accumulation of licorice secondary metabolites. In the PLS-PM equation, red indicates positive correlation, black indicates negative correlation. The path diagram represents the correlations between each pair of variables. “**” denotes highly significant correlation (p ≤ 0.01), and ““ denotes significant correlation (p ≤ 0.05) between the variables. [file Image3.png]

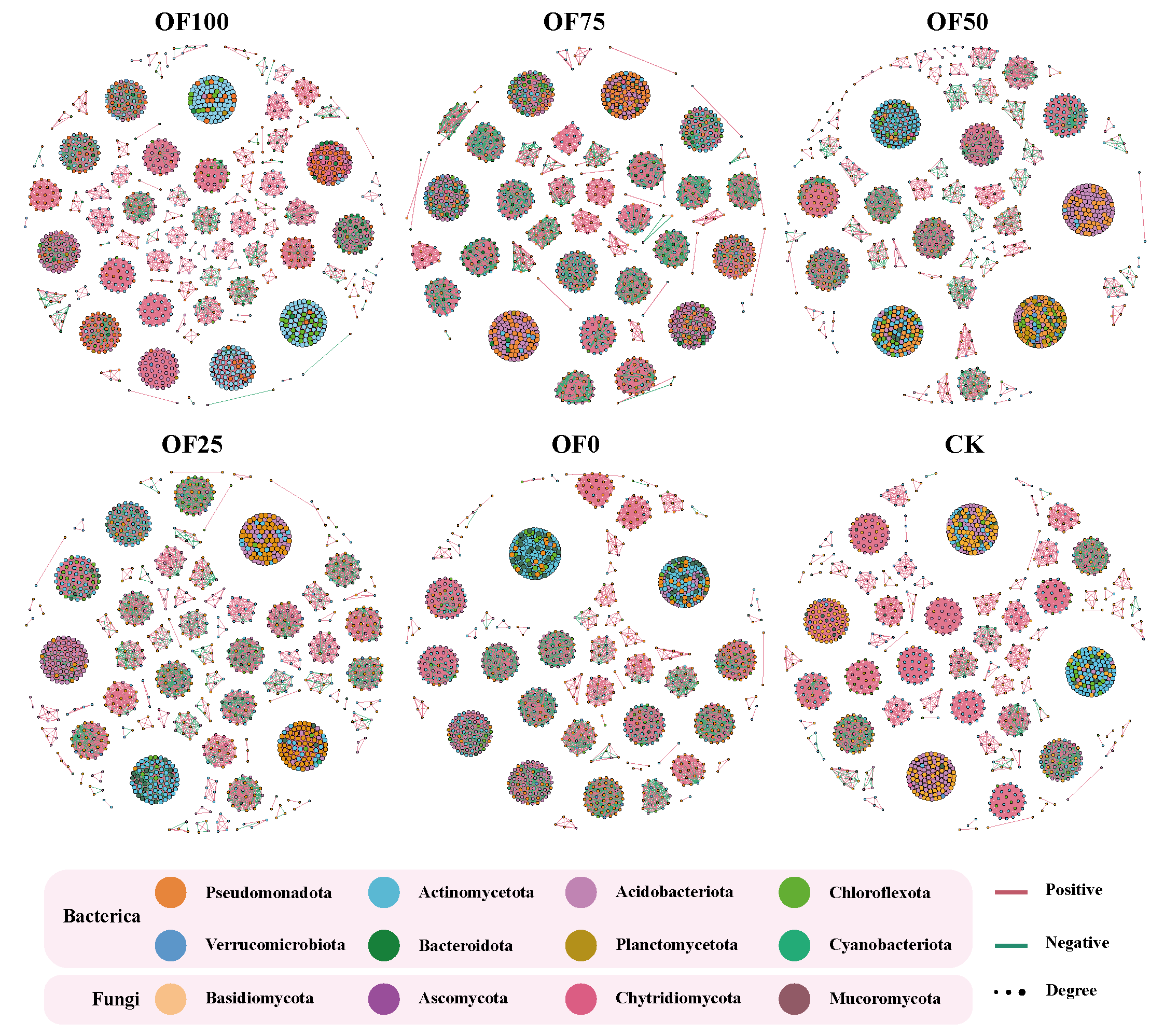

Supplement: Supplementary Figure 4 — Analysis of the co-occurrence network of dominant bacteria and fungi. The co-occurrence network diagrams depict symbiotic patterns between bacterial and fungal species in soil samples from bulk and rhizosphere soils under different fertilization treatments. Edge colors represent positive (purple) and negative (green) correlations. Connections repre-sent strong correlations (Spearman’s r > 0.85) and significant correlations (p ≤ 0.01). Nodes in the co-occurrence networks are colored by phylum level. [file Image4.tif]

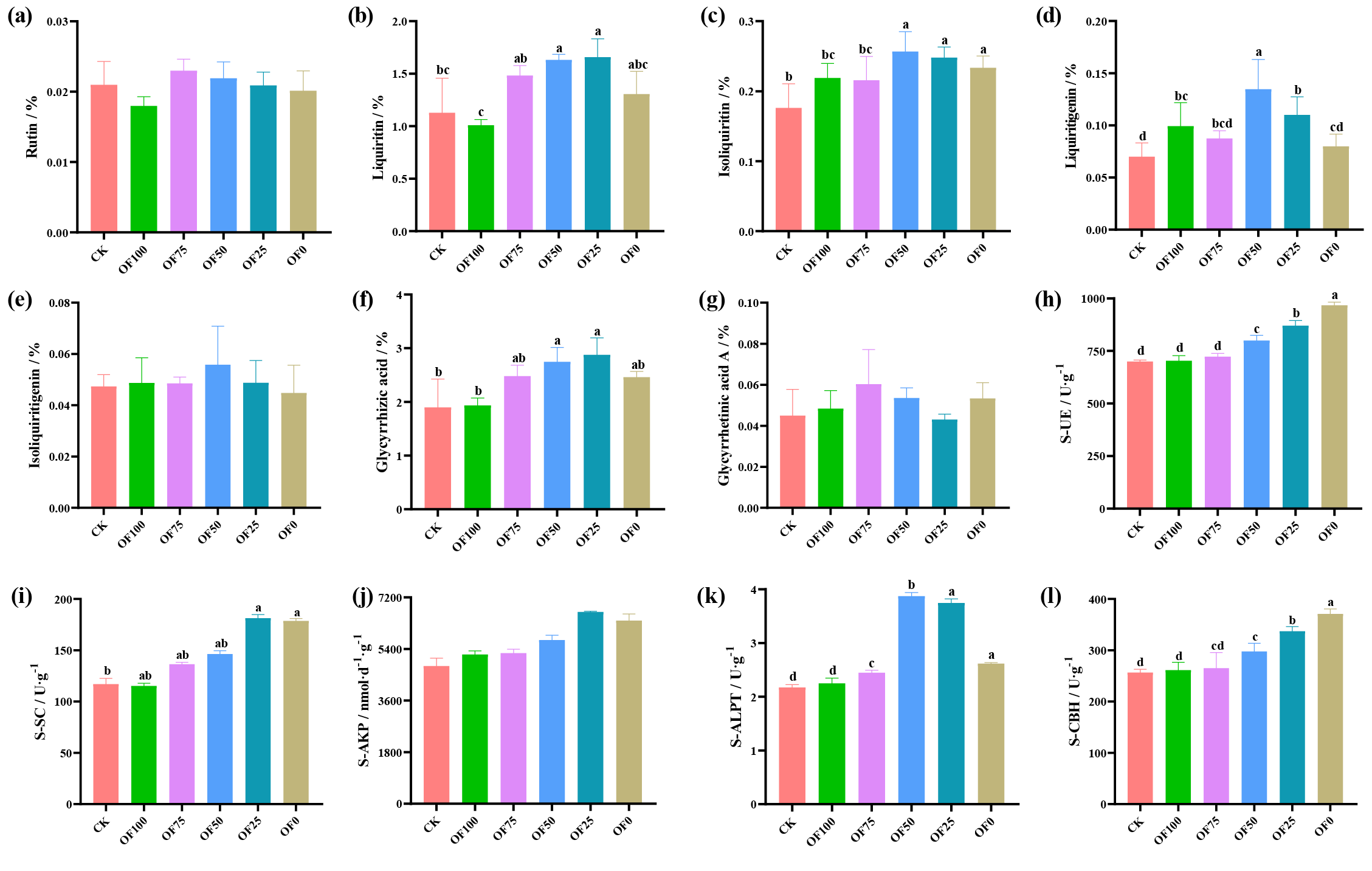

Supplement: Supplementary file 5 [file Figure1.png]

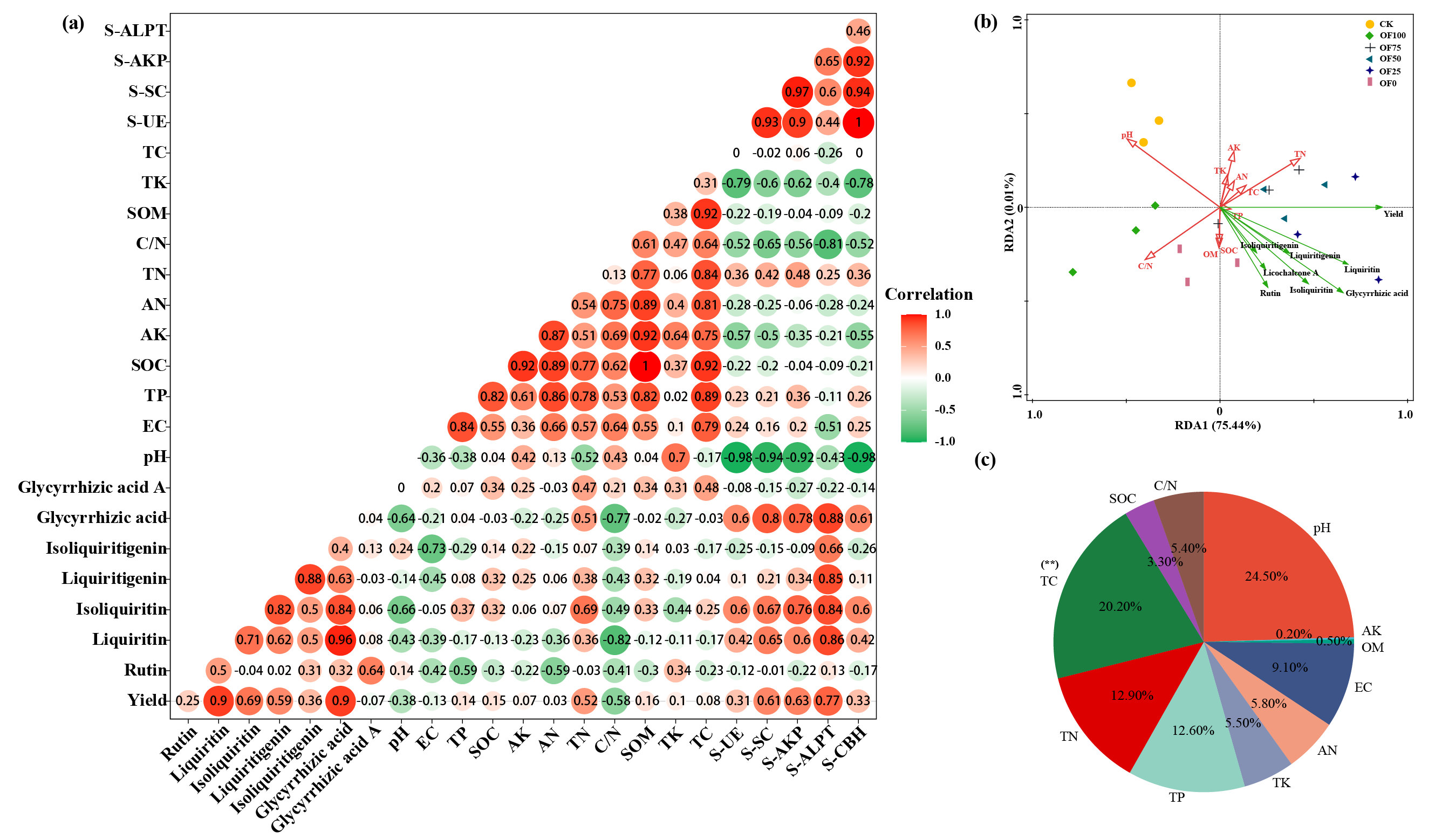

Supplement: Supplementary file 6 [file Figure2.png]

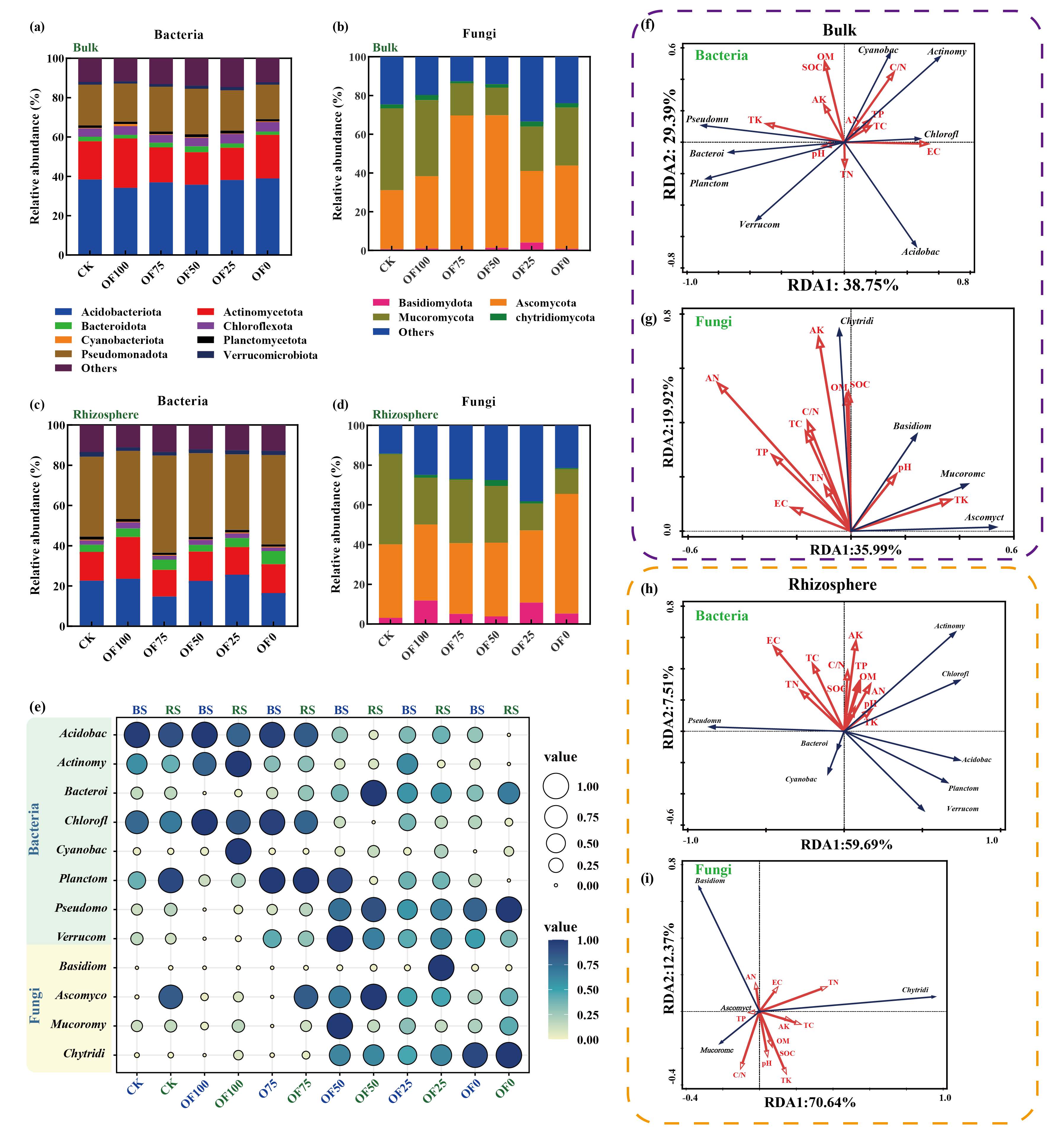

Supplement: Supplementary file 7 [file Figure3.png]

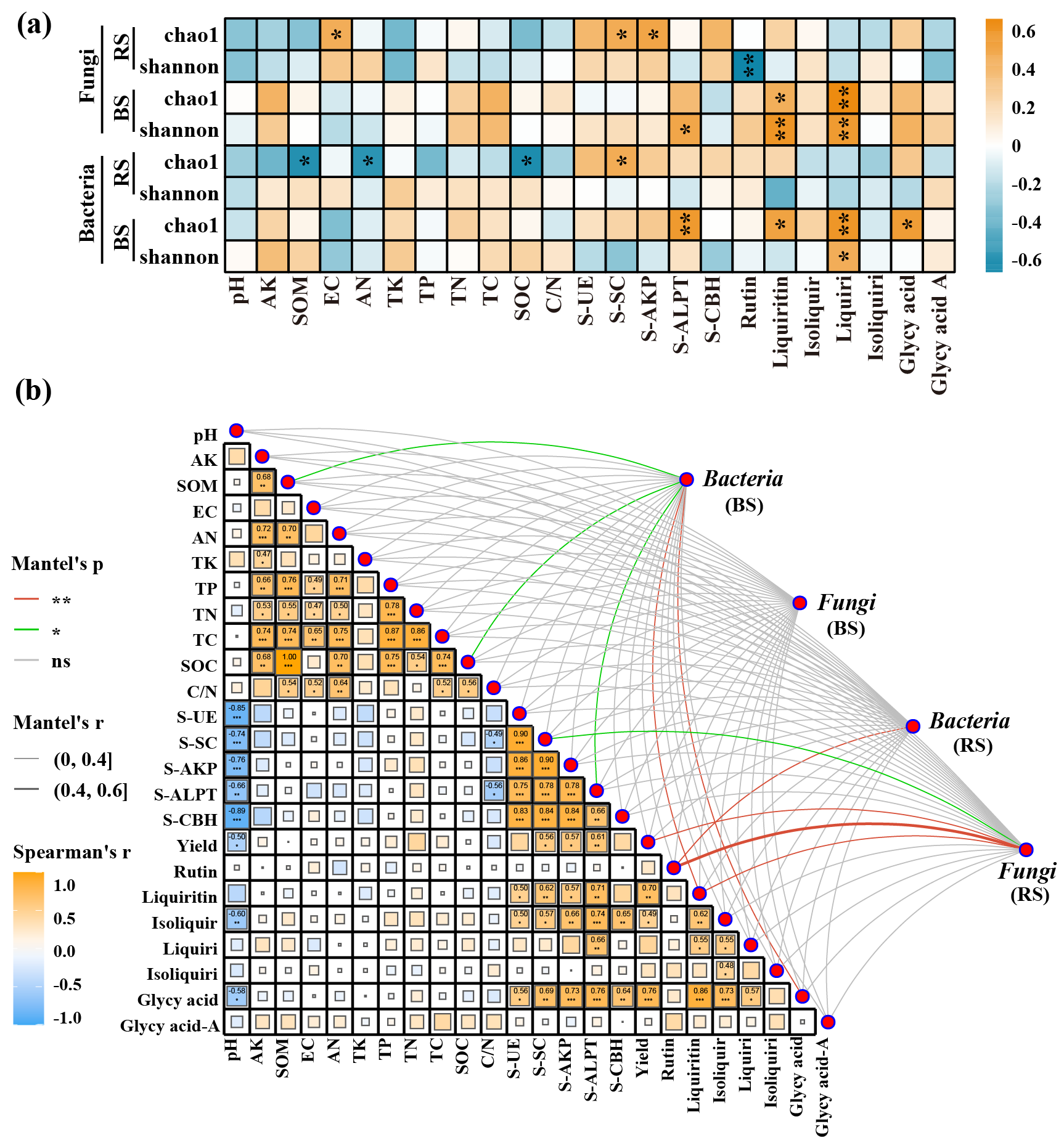

Supplement: Supplementary file 8 [file Figure4.png]
