# Supplementary material for: Mechanistic insights from metagenomics into the early-stage quality improvement of licorice under partial replacement of chemical by organic fertilizers
Source: Front Plant Sci. 2025 Jun 25;16:1613771. doi: 10.3389/fpls.2025.1613771 (PMC12239752; doi:10.3389/fpls.2025.1613771)
Supplement: Supplementary file 9 [file Table1.docx]

**Table S1**

Effects of different fertilizer treatments on the character and yield of *G. uralensis* （ ± s，n=15）

| **Group** | **Root length/cm** | **Root weight/g** | **D1/cm** | **D20/cm** | **Yield/g·m^2^** |
| --- | --- | --- | --- | --- | --- |
| CK | 41.23±1.08a | 8.13±0.56d | 6.79±0.38c | 5.74±0.78a | 101.47±7.01d |
| OF100 | 42.56±1.03a | 8.73±0.49cd | 6.96±0.38bc | 5.11±0.44a | 109.11±6.16cd |
| OF75 | 42.75±2.25a | 10.87±0.82ab | 8.50±0.11a | 5.72±0.21a | 135.84±10.27ab |
| OF50 | 41.15±1.46a | 10.60±0.47abc | 7.90±0.41ab | 5.93±0.65a | 132.50±5.93abc |
| OF25 | 41.88±2.93a | 12.28±0.38a | 8.91±0.84a | 5.97±0.33a | 153.53±4.69a |
| OF0 | 40.03±1.17a | 9.14±1.42bcd | 7.34±0.14bc | 5.11±0.35a | 114.24±17.81bcd |

Different lowercase letters indicate significant differences among treatments (*p* < 0.05) according to Duncan’s Multiple Range Test (DMRT).

**Table S2**

Soil physical and chemical properties of different fertilizer treatments (** ± s，n=3)

| **Group** | **pH** | **AK/mg·kg^-1^** | **AN/mg·kg^-1^** | **TK/g·kg^-1^** | **TN/g·kg^-1^** | **TP/g·kg^-1^** | **TC/g·kg^-1^** | **SOM/g·kg^-1^** | **SOC/g·kg^-1^** | **EC/ms·m^-1^** |
| --- | --- | --- | --- | --- | --- | --- | --- | --- | --- | --- |
| CK | 8.51±0.02a | 132±25.5c | 69±7.4b | 19.97±0.27a | 1.01±0.04a | 0.48±0.38b | 33.1±3.74b | 16.4±1.68b | 9.52±0.97b | 13.0±1.97ab |
| OF100 | 8.40±0.12ab | 231±35.9a | 83±4.6a | 20.01±0.18a | 1.27±0.19a | 0.59±0.28a | 46.2±5.57a | 19.5±0.95a | 11.37±0.54a | 14.73±1.33ab |
| OF75 | 8.37±014ab | 200±19.8ab | 75±6.1ab | 20.26±0.17a | 1.32±0.09a | 0.55±0.19ab | 44.7±5.28ab | 18.7±0.66ab | 10.83±0.40ab | 14.4±0.26ab |
| OF50 | 8.26±0.10bc | 163±28.4bc | 70±3.7ab | 19.81±0.85a | 1.24±0.15a | 0.51±0.47ab | 37.8±3.13ab | 17.9±0.88ab | 10.39±0.52ab | 12.4±0.65a |
| OF25 | 8.12±0.06cd | 157±13.8bc | 75±2.8ab | 19.87±0.33a | 1.25±0.07a | 0.55±0.25ab | 38.7±1.55ab | 17.8±0.59ab | 10.3±0.36ab | 13.8±1.56ab |
| OF0 | 7.94±0.09d | 128±15.9c | 72±5.3ab | 19.65±0.27a | 1.28±0.16a | 0.56±0.68ab | 35.4±3.48ab | 17.6±1.65ab | 10.23±0.96ab | 14.9±0.54b |

Different letters indicate a statistically significant difference between treatment methods (*p* < 0.05) according to Duncan’s Multiple Range Test (DMRT).

**Table S3**

The situation of *G. uralensis* by CRITIC weight method

| **Indicator** | **Variability** | **Conflict** | **Information** | **Weight/%** |
| --- | --- | --- | --- | --- |
| Yield | 0.376 | 3.377 | 1.269 | 11.23 |
| Rutin | 0.354 | 5.001 | 1.770 | 15.66 |
| Glycyrrhizin | 0.413 | 2.715 | 1.120 | 9.91 |
| Isoglycyrrhizin | 0.356 | 3.406 | 1.212 | 10.73 |
| Glycyrrhizin | 0.360 | 3.461 | 1.245 | 11.02 |
| Isoglycyrrhizin | 0.333 | 3.929 | 1.309 | 11.58 |
| Glycyrrhizic acid | 0.415 | 2.909 | 1.208 | 10.69 |
| Glycyrrhetinic acid A | 0.353 | 6.145 | 2.168 | 19.19 |

**Table S4**

Comprehensive analysis of the quality of medicinal materials in different treatments

| **Treatment Group** | **Composite** | **Score Rank** |
| --- | --- | --- |
| CK | 18.73 | 6 |
| OF100 | 24.78 | 5 |
| OF75 | 57.29 | 3 |
| OF50 | 83.99 | 1 |
| OF25 | 67.96 | 2 |
| OF0 | 42.13 | 4 |

**Table S5**

Abundance of dominant bacteria in **Bulk soils** under different fertilization regimes at phylum, class, and order levels

| ***Phyla*** | ***Class*** | ***Order*** | **BCK** | **BOF100** | **BOF75** | **BOF50** | **BOF25** | **BOF0** | **F** | **P** |
| --- | --- | --- | --- | --- | --- | --- | --- | --- | --- | --- |
| ***Acidobac*** |  |  | 38.38±0.26a | 34.11±4.97a | 36.96±1.08a | 35.67±2.65a | 38.15±5.12a | 38.91±3.43a | 0.648 | 0.669 |
|  | *Blastocatellia* |  | 46.03±7.39a | 39.17±3.64a | 40.52±1.81a | 41.11±7.93a | 42.32±3.41a | 43.53±3.59a | 0.447 | 0.807 |
|  |  | *Pyrinomonadale* | 95.94±0.27a | 95.75±0.25a | 95.73±0.03a | 95.41±0.60a | 95.54±0.33a | 95.87±0.26a | 0.711 | 0.626 |
|  | *Terriglobia* |  | 3.83±2.75a | 5.60±0.28a | 6.03±0.06a | 6.55±0.99a | 6.33±0.46a | 3.68±2.59a | 1.188 | 0.371 |
|  |  | *Terriglobia* | 20.19±1.16a | 20.33±0.66a | 19.57±0.05a | 19.13±1.53a | 18.50±0.53a | 20.13±0.79a | 1.228 | 0.355 |
|  | *Verrucomicrob* |  | 32.85±23.25a | 51.15±3.49a | 49.67±2.38a | 48.56±7.11a | 46.53±2.87a | 31.57±22.19a | 0.837 | 0.548 |
|  |  | *Vicinamibactera* | 95.81±0.63a | 96.96±0.53a | 96.48±0.52a | 95.59±0.45a | 95.04±1.41a | 96.67±0.47a | 1.868 | 0.174 |
| ***Actinomy*** |  |  | 19.47±3.21bc | 25.12±3.43a | 17.75±0.70bc | 16.54±1.03c | 16.34±2.50c | 22.18±3.43ab | **4.750** | **0.013** |
|  | *Acidimicrobiia* |  | 4.14±0.47b | 4.27±0.41ab | 4.72±0.24ab | 5.06±0.14a | 4.65±0.38ab | 4.35±0.16ab | 2.228 | 0.119 |
|  |  | *Acidimicrobiale* | 83.51±0.70b | 85.91±0.79a | 83.41±0.44b | 82.97±0.26b | 82.69±0.88b | 83.81±0.65b | **6.124** | **0.005** |
|  | *Actinomycetes* |  | 44.22±8.91a | 41.20±1.31a | 39.26±1.26a | 40.97±1.05a | 39.89±6.13a | 37.33±1.04a | 0.519 | 0.757 |
|  |  | *Actinomycetales* | 9.75±1.43a | 10.08±0.64a | 9.33±0.57a | 9.37±0.09a | 9.41±0.48a | 9.65±0.03a | 0.330 | 0.885 |
|  |  | *Mycobacterial* | 37.78±9.06a | 41.95±0.64a | 44.63±1.27a | 43.22±0.63a | 44.48±5.64a | 41.84±0.39a | 0.655 | 0.664 |
|  |  | *Streptomycetale* | 3.18±0.81ab | 2.95±0.31b | 3.85±0.07ab | 4.31±0.26a | 3.91±0.61ab | 3.67±0.24ab | 2.414 | 0.098 |
|  | *Thermoleophilia* |  | 39.58±6.88a | 42.97±0.78a | 42.66±0.92a | 41.20±1.29a | 41.06±3.79a | 45.57±0.89a | 0.761 | 0.595 |
|  |  | *Solirubrobacter* | 54.88±2.35ab | 58.62±3.14a | 51.43±0.20b | 52.95±0.28b | 51.67±0.98b | 54.78±0.81ab | **5.003** | **0.010** |
| ***Bacteroi*** |  |  | 2.31±0.39abc | 1.79±0.21bc | 2.39±0.27ab | 3.00±0.44a | 2.20±0.26bc | 1.62±0.11c | **5.269** | **0.009** |
|  | *Bacteroides* |  | 95.68±0.86a | 94.97±0.77ab | 95.72±0.43a | 96.35±0.34a | 95.10±0.63ab | 93.94±0.44b | **3.705** | **0.029** |
|  |  | *Bacteroidales* | 1.28±0.16a | 1.65±0.07a | 1.72±0.29a | 1.71±0.33a | 1.52±0.14a | 1.69±0.03a | 1.463 | 0.272 |
|  |  | *Chitinophagales* | 86.68±3.78a | 84.08±1.36a | 82.03±0.31a | 82.09±3.35a | 83.57±1,03a | 84.94±1.44a | 1.230 | 0.354 |
|  |  | *Cytophagales* | 8.50±3.43a | 9.51±0.90a | 10.82±1.06a | 11.53±2.33a | 10.56±0.30a | 8.52±0.33a | 0.981 | 0.468 |
|  |  | *Flavobacteriale* | 1.57±0.26a | 2.71±0.73a | 2.62±0.92a | 2.07±0.25a | 1.98±0.45a | 2.72±1.39a | 0.749 | 0.602 |
| ***Chlorofl*** |  |  | 4.14±0.59a | 4.46±0.39a | 3.96±0.33a | 4.31±0.58a | 4.83±0.21a | 4.91±0.38a | 1.508 | 0.259 |
|  | *Anaerolineae* |  | 38.32±2.53b | 33.45±1.42b | 35.34±2.73b | 38.86±1.91b | 46.47±5.39a | 36.01±1.93b | **4.808** | **0.012** |
|  |  | *Anaerolineales* | 72.09±2.94ab | 70.73±2.20ab | 67.86±2.76b | 71.81±2.95ab | 76.68±1.76a | 72.75±1.10ab | 2.900 | 0.061 |
|  | *Chloroflexota* |  | 3.21±0.43a | 3.03±0.09a | 3.82±0.12a | 3.64±0.87a | 2.82±0.08a | 2.85±0.08a | 2.114 | 0.134 |
|  |  | *Dehalococcoida* | 2.65±0.16b | 2.93±0.08ab | 2.93±0.09ab | 2.91±0.12ab | 3.29±0.43a | 2.73±0.07b | 2.392 | 0.100 |
| ***Cyanobac*** |  |  | 0.31±0.03a | 1.03±0.69a | 0.31±0.02a | 0.30±0.01a | 0.30±0.03a | 0.32±0.03a | 2.140 | 0.130 |
|  | *Cyanobacteria* |  | 96.06±0.64b | 98.23±1.21a | 95.17±0.35b | 94.97±0.50b | 95.76±0.21b | 96.17±0.48b | **6.453** | **0.004** |
|  |  | *Cyanobacteriale* | 77.48±2.75ab | 86.64±9.03a | 74.03±0.33b | 75.01±1.87b | 75.35±1.24b | 77.25±1.87ab | 2.611 | 0.080 |
| ***Pseudomo*** |  |  | 48.11±4.43ab | 52.03±1.02ab | 46.38±2.16ab | 46.94±2.69a | 49.98±2.44ab | 54.57±0.38b | 2.030 | 0.146 |
|  | *Alphaproteobac* |  | 42.94±2.59abc | 47.12±2.93ab | 41.70±3.37bc | 41.43±1.54bc | 38.98±1.07c | 48.52±1.33a | **4.996** | **0.011** |
|  |  | *Rhizobiales* | 48.63±1.44ab | 43.89±1.58b | 49.87±2.88a | 50.29±1.48a | 47.80±3.60ab | 43.63±0.68b | **3.592** | **0.032** |
|  |  | *Sphingomonada* | 20.41±6.26ab | 25.64±2.80a | 18.42±1.25b | 15.01±0.69b | 20.11±2.42ab | 25.61±0.90a | **5.961** | **0.005** |
|  | *Gammaproteob* |  | 57.04±2.60abc | 52.86±2.94bc | 58.27±3.37ab | 58.52±1.53ab | 60.99±1.07a | 51.45±1.33c | **4.984** | **0.011** |
|  |  | *Burkholderiales* | 77.74±2.79a | 73.69±1.30a | 72.20±3.90a | 69.96±6.91a | 78.75±2.38a | 72.07±2.18a | 1.728 | 0.203 |
|  |  | *Pseudomonadal* | 0.98±0.04a | 1.99±0.18a | 2.67±1.57a | 3.62±2.49a | 1.09±0.14a | 1.21±0.10a | 1.519 | 0.256 |
| ***Plancton*** |  |  | 1.22±0.12abc | 1.18±0.06bc | 1.45±0.04ab | 1.49±0.23a | 1.46±0.10ab | 1.31±0.02c | **3.841** | **0.026** |
|  | *Phycisphaerae* |  | 18.52±1.08bc | 23.07±0.66a | 17.42±1.37c | 17.07±0.22c | 16.51±0.92c | 20.28±0.60b | **15.511** | **0.000** |
|  |  | *Phycisphaerales* | 27.20±2.54ab | 22.71±1.86b | 30.41±0.26a | 32.55±4.83a | 31.08±0.09a | 26.83±0.36ab | **4.638** | **0.014** |
|  | *Planctomycetes* |  | 48.11±4.43a | 52.03±1.02a | 46.37±2.16a | 46.94±2.69a | 49.97±2.44a | 54.57±0.38a | 1.783 | 0.237 |
|  |  | *Pirellulales* | 38.82±0.63abc | 37.55±1.28c | 40.72±1.34ab | 42.03±1.12ab | 43.04±3.5a7 | 37.90±0.93bc | **3.288** | **0.042** |
| ***Verrucom*** |  |  | 1.37±0.22bc | 1.22±0.12c | 1.34±0.03bc | 1.57±0.12ab | 1.70±0.19a | 1.19±0.04c | **4.194** | **0.019** |
|  | *Verrucomicrobi* |  | 98.20±0.07a | 97.84±0.25a | 97.80±0.16a | 97.95±0.34a | 98.31±0.09a | 98.11±0.11a | 2.188 | 0.124 |
|  |  | *Pedosphaerales* | 70.50±2.00b | 69.52±0.74b | 69.87±1.21b | 70.29±0.73b | 75.12±2.27a | 68.04±0.88b | **5.528** | **0.007** |

Different letters indicate a statistically significant difference between treatment methods (*p* < 0.05) according to Duncan’s Multiple Range Test (DMRT). Abbreviations of microbial taxa: Acidobac, Acidobacteriota; Actinomy, Actinomycetota; Bacteroi, Bacteroidota; Chlorofl, Chloroflexota; Cyanobac, Cyanobacteriota; Planctom, Planctomycetota; Pseu-domo, Pseudomonadota; Verrucom, Verrucomicrobiota.

**Table S6**

The abundance of dominant bacteria in **Rhizosphere** **soils** under different fertilization regimes at phylum, class, and order levels

| ***Phyla*** | ***Class*** | ***Order*** | **RCK** | **ROF100** | **ROF75** | **ROF50** | **ROF25** | **ROF0** | **F** | **P** |
| --- | --- | --- | --- | --- | --- | --- | --- | --- | --- | --- |
| ***Acidobac*** |  |  | 22.51±5.36ab | 25.56±2.19ab | 14.76±1.55c | 22.40±4.33abc | 25.64±1.72a | 16.47±0.93bc | **3.760** | **0.028** |
|  | *Blastocatellia* |  | 41.89±2.32a | 40.32±3.63ab | 32.61±1.04b | 47.01±1.71a | 43.80±5.44a | 42.92±4.01a | **3.934** | **0.024** |
|  |  | *Pyrinomonadales* | 95.62±0.20a | 95.62±0.16a | 94.73±0.21b | 95.82±0.20a | 95.73±0.24a | 95.63±0.18a | **7.827** | **0.002** |
|  | *Terriglobia* |  | 6.43±0.38ab | 5.56±0.14b | 6.98±0.13a | 5.98±0.39b | 5.89±0.61b | 6.42±0.23ab | **4.039** | **0.022** |
|  |  | *Terriglobia* | 20.30±0.79a | 21.85±0.63a | 20.71±0.35a | 21.88±0.36a | 21.52±1.30a | 21.66±0.38a | 1.681 | 0.213 |
|  | *Verrucomicrobiae* |  | 45.47±4.71a | 48.63±4.22a | 50.84±0.26a | 42.99±1.41a | 45.15±4.70a | 44.63±3.50a | 1.312 | 0.323 |
|  |  | *Vicinamibacterales* | 97.47±0.45a | 97.74±0.10a | 97.17±0.15a | 97.25±0.33a | 97.75±0.20a | 97.43±0.23a | 1.583 | 0.238 |
| ***Actinomy*** |  |  | 14.26±2.43b | 20.75±0.74a | 13.26±0.88b | 14.63±0.79b | 13.68±0.75b | 14.31±1.26b | **9.360** | **0.001** |
|  | *Acidimicrobiia* |  | 6.00±0.13bc | 6.18±0.25ab | 6.97±0.24a | 7.09±0.20a | 6.83±0.56ab | 5.20±0.60c | **7.377** | **0.002** |
|  |  | *Acidimicrobiales* | 84.70±0.30c | 87.17±0.43a | 87.36±0.06a | 86.22±0.25ab | 85.46±0.29bc | 86.50±1.08ab | **7.833** | **0.002** |
|  | *Actinomycetes* |  | 50.91±5.27ab | 49.01±3.66b | 52.51±1.89ab | 49.36±2.03b | 45.12±3.11b | 59.30±5.34a | **3.099** | **0.050** |
|  |  | *Actinomycetales* | 8.91±2.29ab | 10.00±0.64ab | 9.25±0.13ab | 8.96±0.83ab | 10.53±0.63a | 7.34±0.90b | 1.909 | 0.168 |
|  |  | *Mycobacteriales* | 46.92±4.94ab | 39.08±4.18b | 45.95±0.36ab | 44.83±2.32ab | 41.58±0.92ab | 49.19±3.81a | 2.596 | 0.082 |
|  |  | *Streptomycetes* | 11.53±2.60b | 10.39±1.64b | 11.07±0.74b | 10.53±0.67b | 9.53±0.93b | 17.39±3.36a | **4.317** | **0.018** |
|  | *Thermoleophilia* |  | 34.47±4.79ab | 34.98±3.33ab | 32.33±1.55ab | 35.36±1.49ab | 38.08±2.89a | 28.46±3.67b | 2.106 | 0.135 |
|  |  | *Solirubrobacterales* | 55.71±1.90b | 58.00±0.78ab | 56.86±0.70b | 57.78±0.58ab | 55.39±0.29b | 60.79±2.97a | **3.259** | **0.042** |
| ***Bacteroi*** |  |  | 3.51±0.33b | 4.31±0.92ab | 5.10±0.95ab | 3.33±0.61b | 4.52±0.68ab | 6.59±1.97a | 2.585 | 0.083 |
|  | *Bacteroides* |  | 97.82±0.25a | 98.24±0.37a | 98.59±0.25a | 65.47±45.60a | 98.01±0.40a | 98.85±0.17a | 1.037 | 0.439 |
|  |  | *Bacteroidales* | 1.52±0.15a | 1.24±0.12a | 1.37±0.11a | 1.36±0.14a | 1.38±0.05a | 1.40±0.39a | 0.435 | 0.816 |
|  |  | *Chitinophagales* | 59.57±6.30a | 64.22±12.68a | 62.45±3.38a | 70.46±2.05a | 67.08±4.27a | 57.33±18.05a | 0.499 | 0.772 |
|  |  | *Cytophagales* | 26.87±5.41a | 17.75±5.19a | 23.58±0.49a | 21.50±1.20a | 18.99±0.73a | 31.74±14.76a | 1.186 | 0.372 |
|  |  | *Flavobacteriales* | 9.39±3.56a | 14.46±13.37a | 10.21±3.84a | 4.30±0.70a | 9.60±3.04a | 5.62±1.57a | 0.716 | 0.623 |
| ***Chlorofl*** |  |  | 2.15±0.47b | 2.97±0.37a | 1.89±0.09b | 2.40±0.25ab | 2.31±0.08ab | 1.71±0.39b | **4.043** | **0.022** |
|  | *Anaerolineae* |  | 29.25±2.85ab | 21.35±3.66b | 28.73±0.86ab | 35.16±2.70a | 29.96±2.37ab | 29.84±1.90b | 2.454 | 0.094 |
|  |  | *Anaerolineales* | 55.33±4.42b | 53.73±1.28b | 56.83±1.51b | 65.09±3.11a | 56.59±3.88b | 54.09±3.27b | **3.562** | **0.033** |
|  | *Chloroflexota* |  | 4.71±0.91a | 3.69±0.64a | 4.87±0.16a | 4.13±0.38a | 4.52±0.32a | 5.15±1.07a | 1.239 | 0.350 |
|  |  | *Dehalococcoidales* | 2.79±0.42a | 2.57±0.66a | 3.16±0.31a | 3.21±0.20a | 2.82±0.06a | 3.04±0.68a | 0.610 | 0.694 |
| ***Cyanobac*** |  |  | 0.37±0.04ab | 0.33±0.04b | 0.28±0.01ab | 0.31±0.01b | 0.47±0.10a | 0.42±0.03ab | **3.237** | **0.044** |
|  | *Cyanobacteria* |  | 69.31±9.22bc | 87.12±4.84a | 57.27±0.94cd | 79.04±3.01ab | 59.87±10.40cd | 53.66±4.23d | **8.602** | **0.001** |
|  |  | *Cyanobacterial* | 74.97±2.00a | 79.68±4.07a | 74.51±0.41a | 75.92±1.80a | 76.92±0.69a | 76.08±1.42a | 1.536 | 0.251 |
| ***Pseudomo*** |  |  | 39.84±6.50bc | 33.89±2.21c | 48.28±1.09a | 41.66±3.59abc | 37.47±1.30bc | 44.40±2.05ab | **4.627** | **0.014** |
|  | *Alphaproteobacteria* |  | 52.46±2.00a | 51.69±2.55a | 47.89±1.16a | 49.07±1.38a | 47.61±0.60a | 52.53±3.10a | 2.635 | 0.079 |
|  |  | *Rhizobiales* | 48.57±1.69a | 49.70±2.47a | 48.02±2.16a | 49.42±1.17a | 45.37±2.96a | 47.51±0.55a | 1.233 | 0.353 |
|  |  | *Sphingomonadales* | 13.07±2.95b | 17.34±1.38a | 14.29±1.45ab | 13.08±0.64b | 18.00±1.09a | 16.26±1.18ab | **3.571** | **0.033** |
|  | *Gammaproteobacteria* |  | 47.52±2.00a | 48.29±2.55a | 52.09±1.16a | 50.90±1.38a | 52.37±0.60a | 47.45±3.10a | 2.642 | 0.078 |
|  |  | *Burkholderiales* | 37.89±9.12ab | 47.27±9.69a | 27.60±1.88b | 26.26±3.47b | 37.07±3.57ab | 31.36±7.65ab | 2.788 | 0.068 |
|  |  | *Pseudomonadales* | 3.92±0.81ab | 3.43±0/89b | 4.84±0.64ab | 3.97±1.13ab | 2.82±0.41b | 6.41±1.83a | 2.856 | 0.063 |
| ***Plancton*** |  |  | 1.51±0.26a | 1.29±0.11a | 1.18±0.07a | 1.23±0.10a | 1.32±0.03a | 1.18±0.16a | 1.484 | 0.266 |
|  | *Phycisphaerae* |  | 17.57±0.89bc | 20.71±1.24a | 17.33±0.22bc | 18.17±0.24abc | 19.75±0.19ab | 15.82±2.56c | **4.125** | **0.021** |
|  |  | *Phycisphaerales* | 27.69±2.65ab | 23.78±1.91b | 29.78±0.12a | 28.00±0.50ab | 27.42±0.18ab | 31.05±2.81a | **3.987** | **0.023** |
|  | *Planctomycetes* |  | 44.06±4.59a | 54.76±2.99a | 51.12±1.92a | 52.72±0.41a | 51.72±0.27a | 48.49±8.22a | 1.667 | 0.217 |
|  |  | *Pirellulales* | 35.47±0.75c | 36.46±0.42bc | 40.31±0.32a | 37.61±0.79bc | 38.86±1.55ab | 35.96±1.81c | **5.807** | **0.006** |
| ***Verrucom*** |  |  | 2.47±0.53a | 1.78±0.14b | 1.75±0.05b | 1.91±0.10ab | 2.05±0.08ab | 2.10±0.25ab | 2.209 | 0.121 |
|  | *Verrucomicrobiae* |  | 98.67±0.30a | 98.47±0.15ab | 98.23±0.01ab | 98.05±0.38b | 98.49±0.08ab | 98.53±0.07ab | 2.285 | 0.112 |
|  |  | *Pedosphaerales* | 52.40±12.92a | 49.06±5.09a | 38.20±2.30ab | 43.60±3.67ab | 53.23±0.69a | 32.63±3.57b | **3.627** | **0.031** |

Different letters indicate a statistically significant difference between treatment methods (*p* < 0.05) according to Duncan’s Multiple Range Test (DMRT). Abbreviations of microbial taxa: Acidobac, Acidobacteriota; Actinomy, Actinomycetota; Bacteroi, Bacteroidota; Chlorofl, Chloroflexota; Cyanobac, Cyanobacteriota; Planctom, Planctomycetota; Pseu-domo, Pseudomonadota; Verrucom, Verrucomicrobiota.

**Table S7**

Abundance of dominant fungi in **Bulk soils** under different fertilization regimes at phylum, class, and order levels

| ***Phyla*** | ***Class*** | ***Order*** | **BCK** | **BOF100** | **BOF75** | **BOF50** | **BOF25** | **BOF0** | **F** | **P** |
| --- | --- | --- | --- | --- | --- | --- | --- | --- | --- | --- |
| ***Basidiom*** |  |  | 0.785±0.11a | 1.01±0.05a | 0.694±0.07a | 1.379±0.07a | 4.122±0.22b | 0.89±0.04a | **4.306** | **0.019** |
|  | *Agaricomycetes* |  | 48.58±3.82b | 58.19±3.51b | 62.43±10.09b | 51.48±3.08b | 53.43±7.96a | 67.07±7.33a | **3.932** | **0.024** |
|  |  | *Agarica* | 9.71±3.41a | 17.16±3.11a | 10.71±3.42a | 15.04±6.02a | 9.39±5.55a | 11.84±2.18a | 1.121 | 0,400 |
| ***Ascomyco*** |  |  | 30.31±5.06a | 37.30±8.04a | 69.04±18.40a | 68.45±21.73a | 36.90±6.65a | 42.94±6.86a | 1.537 | 0.251 |
|  | *Dothideomycetes* |  | 14.11±1.08c | 10.69±2.86c | 41.22±21.04ab | 52.80±11.41a | 29.78±4.41abc | 23.56±10.67bc | **4.378** | **0.017** |
|  |  | *Pleosporales* | 50.08±13.92b | 56.40±7.79b | 84.14±4.12a | 87.60±1.93a | 70.63±9.33ab | 67.08±16.33ab | **4.184** | **0.020** |
|  | *Sordariomycetes* |  | 13.51±1.33a | 25.03±7.08a | 21.45±12.01a | 14.04±2.32a | 14.44±1.68a | 13.40±0.41a | 0.127 | 0.983 |
|  |  | *Hypocreales* | 38.94±0.62a | 35.06±2.27a | 34.77±10.56a | 35.17±2.51a | 38.22±11.24a | 38.63±11.08a | 0.126 | 0.967 |
| ***Mucoromy*** |  |  | 42.30±6.49a | 39.31±6.33ab | 16.64±2.73ab | 14.29±3.39b | 22.99±6.01ab | 29.93±8.26ab | 2.359 | 0.104 |
|  | *Glomeromycetes* |  | 85.04±5.72a | 90.26±1.99a | 83.73±6.90a | 85.50±1.49a | 65.64±22.04a | 83.89±4.14a | 1.485 | 0.266 |
|  |  | *Diversisporales* | 4.45±0.59a | 4.34±1.29a | 4.87±0.76a | 5.67±0.61a | 6.98±2.40a | 6.35±1.29a | 1.322 | 0.319 |
|  |  | *Glomerales* | 1.94±0.41a | 1.82±0.69ab | 1.33±0.51ab | 1.78±0.04ab | 2.57±0.14b | 2.25±0.05ab | 2.092 | 0.137 |
| ***Chytridi*** |  |  | 2.01±0.84ab | 2.27±0.27a | 0.94±0.03b | 35.45±15.28ab | 30.67±8.24ab | 24.75±2.65b | 2.177 | 0.125 |
|  | *Chytridiomycetes* |  | 99.67±0.13a | 98.72±0.87a | 99.77±0.03a | 98.55±1.10a | 99.32±0.26a | 99.17±0.87a | 1.043 | 0.437 |
|  |  | *Spizellomycetales* | 53.42±9.21ab | 44.30±15.09ab | 58.92±12.57ab | 69.66±12.32a | 41.10±7.19b | 35.87±4.78b | 2.693 | 0.074 |

Different letters indicate a statistically significant difference between treatment methods (*p* < 0.05) according to Duncan’s Multiple Range Test (DMRT). Abbreviations of microbial taxa: Basidiom, Basidiomycota; Ascomyco, Ascomycota; Mucoromy, Mucoromycota; Chytridi, Chytridiomycota.

**Table S8**

The abundance of dominant fungi in **Rhizosphere soils** under different fertilization regimes at the level of phylum, class, and order

| ***Phyla*** | ***Class*** | ***Order*** | **RCK** | **ROF100** | **ROF75** | **ROF50** | **ROF25** | **ROF0** | **F** | **P** |
| --- | --- | --- | --- | --- | --- | --- | --- | --- | --- | --- |
| ***Basidiom*** |  |  | 3.15±0.18b | 11.88±0.36b | 5.12±0.34b | 3.88±0.03b | 10.80±0.58a | 5.34±0.34b | **3.900** | **0.025** |
|  | *Agaricomycetes* |  | 0.34±0.15b | 0.45±0.15b | 0.44±0.37b | 0.62±0.31b | 30.21±11.20a | 0.45±0.17b | **3.932** | **0.024** |
|  |  | *Agarica* | 17.86±2.88a | 14.33±2.12ab | 14.91±3.01ab | 9.16±0.68bc | 7.61±2.49c | 12.93±2.72abc | **4.806** | **0.012** |
| ***Ascomyco*** |  |  | 36.99±0.99b | 38.24±0.53ab | 35.68±0.46b | 37.08±0.09b | 36.30±0.04b | 60.18±1.12a | **4.418** | **0.016** |
|  | *Dothideomycetes* |  | 52.39±8.59ab | 31.63±6.72c | 40.63±4.11bc | 41.56±6.16bc | 52.34±2.15ab | 57.70±2.11a | **6.202** | **0.005** |
|  |  | *Pleosporales* | 52.03±1.61a | 49.29±12.83a | 46.423.05a | 50.61±7.55a | 64.25±7.74a | 53.75±12.58a | 0.842 | 0.545 |
|  | *Sordariomycetes* |  | 26.99±14.53a | 23.99±2.33a | 30.91±1.44a | 22.46±4.53a | 16.46±0.52a | 32.23±1.68a | 1.685 | 0.213 |
|  |  | *Hypocreales* | 59.06±4.78b | 50.07±4.34b | 56.07±6.09b | 59.15±3.09b | 59.50±5.88b | 72.56±4.58a | **4.524** | **0.015** |
| ***Mucoromy*** |  |  | 45.42±0.93a | 23.58±0.30a | 31.86±0.27a | 28.60±0.05a | 13.63±0.04a | 12.53±0.03a | 1.492 | 0.264 |
|  | *Glomeromycetes* |  | 95.39±2.01a | 92.47±0.83ab | 91.92±0.42ab | 86.99±5.22abc | 80.17±4.71c | 85.75±4.84bc | **4.472** | **0.013** |
|  |  | *Diversisporales* | 5.30±1.82a | 5.63±1.96a | 1.60±0.29b | 4.00±0.70ab | 3.26±0.62ab | 2.05±0.49b | **3.925** | **0.024** |
| ***Chytridi*** |  |  | 0.25±0.02a | 1.43±0.09ab | 0.47±0.02a | 2.95±0.83b | 1.10±0.28b | 0.48±0.06ab | 2.225 | 0.119 |
|  | *Chytridiomycetes* |  | 99.86±0.08a | 99.79±0.08a | 99.96±0.01a | 99.94±0.02a | 99.79±0.13a | 99.90±0.04a | 2.198 | 0.122 |
|  |  | *Spizellomycetales* | 79.08±2.65a | 73.13±3.69a | 77.82±3.96a | 79.00±1.44a | 78.45±1.65a | 75.96±3.23a | 1.246 | 0.347 |

Different letters indicate a statistically significant difference between treatment methods (*p* < 0.05) according to Duncan’s Multiple Range Test (DMRT). Abbreviations of microbial taxa: Basidiom, Basidiomycota; Ascomyco, Ascomycota; Mucoromy, Mucoromycota; Chytridi, Chytridiomycota.

**Table S9**

Topological properties of co-occurrence networks of soil bacterial and fungal communities

| **Microbes** | **Nodes** | **Edges** | **Average degree** | **Connected Components** | **Density** | **Modularity** |
| --- | --- | --- | --- | --- | --- | --- |
| CK | 1145 | 31034 | 54208 | 81 | 0.047 | 0.821 |
| OF100 | 1144 | 27426 | 47948 | 58 | 0.042 | 0.889 |
| OF75 | 1196 | 21936 | 36682 | 102 | 0.031 | 0.901 |
| OF50 | 1144 | 27426 | 47948 | 76 | 0.042 | 0.889 |
| OF25 | 1102 | 21851 | 39657 | 93 | 0.036 | 0.866 |
| OF0 | 1156 | 39817 | 68888 | 65 | 0.06 | 0.776 |
